# Phosphatase-independent activity of smooth-muscle calcineurin orchestrates a gene expression program leading to hypertension

**DOI:** 10.1371/journal.pbio.3003163

**Published:** 2025-05-14

**Authors:** Paula Sofía Yunes-Leites, Yilin Sun, Sara Martínez-Martínez, Álvaro Alfayate, Marta Toral, María José Méndez-Olivares, Ángel Colmenar, Ana Isabel Torralbo, Dolores López-Maderuelo, Sergio Mateos-García, David N. Cornfield, Jesús Vázquez, Juan Miguel Redondo, Miguel R. Campanero

**Affiliations:** 1 Gene Regulation in Cardiovascular Remodeling and Inflammation Group, Centro Nacional de Investigaciones Cardiovasculares (CNIC), Madrid, Spain; 2 Centro de Investigación Biomédica en Red de Enfermedades Cardiovasculares (CIBERCV), Madrid, Spain; 3 Tissue and Organ Homeostais Program, Centro de Biología Molecular Severo Ochoa (CBM), Consejo Superior de Investigaciones Científicas–Universidad Autónoma de Madrid, Madrid, Spain; 4 Cardiovascular Proteomics Laboratoy, CNIC, Madrid, Spain; 5 Department of Pediatrics, Center for Excellence in Pulmonary Biology, Stanford University School of Medicine, Stanford, California, United States of America; Columbia University, UNITED STATES OF AMERICA

## Abstract

Angiotensin-II (Ang-II) drives pathological vascular wall remodeling in hypertension and abdominal aortic aneurysm (AAA) through mechanisms that are not completely understood. Previous studies showed that the phosphatase activity of calcineurin (Cn) mediates Ang-II-induced AAA, but the cell type involved in the action of Cn in AAA formation remained unknown. Here, by employing newly created smooth muscle cell (SMC)-specific and endothelial cell (EC)-specific Cn-deficient mice (SM-Cn^−/−^ and EC-Cn^−/−^ mice), we show that Cn expressed in SMCs, but not ECs, was required for Ang-II-induced AAA. Unexpectedly, SMC Cn also played a structural role in the early onset and maintenance of Ang-II-induced hypertension, independently of its known phosphatase activity. Among the signaling pathways activated by Ang-II, Cn signaling is essential in SMCs, as nearly 90% of the genes regulated by Ang-II in the aorta required Cn expression in SMCs. Cn orchestrated, independently of its enzymatic activity, the induction by Ang-II of a transcriptional program closely related to SMC contractility and hypertension. Cn deletion in SMCs, but not its pharmacological inhibition, impaired the regulation of arterial contractility. Among the genes whose regulation by Ang-II required Cn expression but not its phosphatase activity, we discovered that *Serpine1* was critical for Ang-II-induced hypertension. Indeed, pharmacological inhibition of PAI-1, the protein encoded by *Serpine1*, impaired SMCs contractility and readily regressed hypertension. Mechanistically, *Serpine1* induction was mediated by Smad2 activation via the structural role of Cn. These findings uncover an unexpected role for Cn in vascular pathophysiology and highlight PAI-1 as a potential therapeutic target for hypertension.

## Abstract 2

Calcineurin is a phosphatase that has been implicated in angiotensin-II-induced abdominal aortic aneurysms (AAA). This study reveals a novel, phosphatase-independent mechanism by which Calcineurin contributes to AAA, dependent on its transcriptional reprogramming of smooth muscle cells.

## Introduction

Arteries are complex structures composed of three distinct layers—the intima, media, and adventitia—each of which plays a unique physiological role. The intima is a single layer of endothelial cells (ECs), whereas the adventitia is a composite of various cell types and extracellular matrix (ECM) components. The media comprises multiple layers of ECM and vascular smooth muscle cells (VSMCs), whose contractile ability provides the basis of the overall contractility of the vessel. However, changes in hemodynamic forces or molecular signaling pathways can lead to pathological vascular wall remodeling (VWR), resulting in destabilization of the arterial wall and potentially leading to outcomes such as aortic aneurysm (AA) or arterial hypertension.

AA is a progressive and abnormal enlargement and weakening of the aortic wall that can eventually result in aortic dissection or rupture, accounting for 1%–2% of all deaths in industrialized countries. Before dissection or rupture, AA is often clinically silent, and there is therefore a clear need for improved techniques for early diagnosis. Key features of AA include upregulation of VSMC proliferation, migration, and apoptosis, and the repression in these cells of genes related to quiescence and contractility [1].

Hypertension is one of the most common chronic diseases, affecting more than 1 billion people worldwide. Hypertension is considered a multiorgan outcome, with the underlying cause identified in only 5% of patients [2]. Blood pressure is determined by the contractile state of the arteries, which can be dysregulated by a variety of VSMC-mediated processes, including hyperplasia, hyperproliferation, and increased contractility [3,4].

VWR is triggered by angiotensin-II (Ang-II), a key effector of the renin-angiotensin system. In abdominal aortic aneurysm (AAA), Ang-II activates pro-mitogenic and pro-apoptotic pathways, increases ECM deposition, and induces cell migration and inflammation [5,6]. While the importance of Ang-II in VWR is broadly accepted, the underlying molecular mechanisms are incompletely understood. Ang-II exerts its pathological effects by binding to its type 1 receptor (AT1R), which activates a variety of signaling pathways, including those mediated by increased cytosolic [Ca^2+^], activation of the PKC-dependent tyrosine kinase cascade, NADPH-dependent ROS production, and activation of JAK/STAT, AKT, and MAPK kinases [6].

One of the key effectors activated by the increase in cytosolic [Ca^2+^] is the ubiquitously expressed Ca^2+^/calmodulin-dependent serine/threonine phosphatase calcineurin (Cn). Cn, comprising a catalytic subunit (CnA) and a regulatory subunit (CnB), plays a key role in the development of pathological VWR by mediating Ang-II-induced VSMC migration [7] and senescence [8], regulating endothelial nitric oxide synthase activity and endothelial barrier function in ECs [9,10], and mediating fibroblast migration and collagen secretion [11]. Inhibition of Cn activity with cyclosporine A (CsA) diminishes Ang-II-triggered neointima formation and AAA development, which is also inhibited by lentiviral expression of Cn-blocking peptides [7,12]. Despite the importance of Cn in pathological VWR, knowledge is lacking about the relative contribution of Cn expressed in the different vascular cell types to the development of vascular disease.

## Results

### Smooth muscle Cn is required for AAA formation

**Fig 1:**
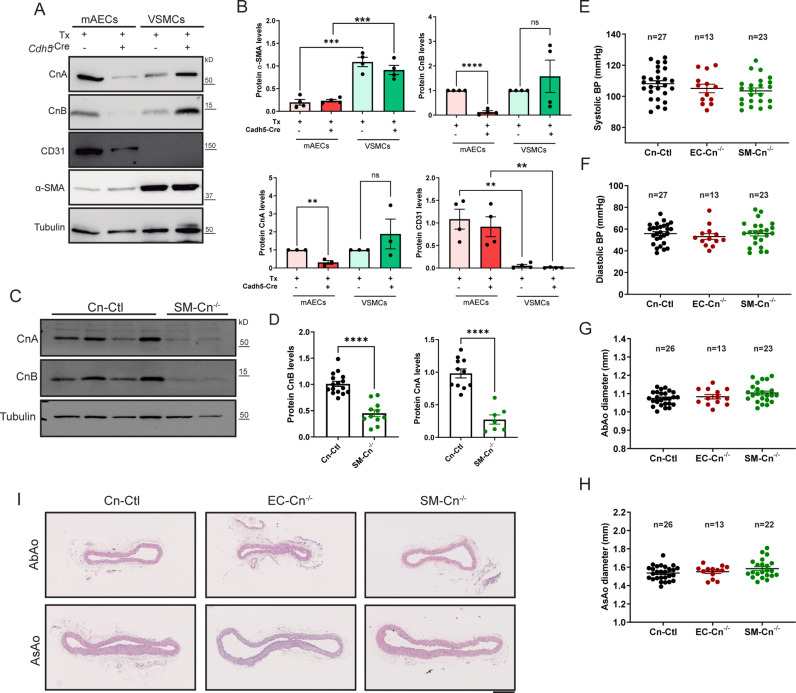
Conditional calcineurin (Cn) deletion causes no evident symptoms in the absence of angiotensin-II (Ang-II). (**A**) Representative immunoblot analysis of CnA, CnB, CD31, α-SMA, and tubulin (loading control) in mouse aortic endothelial cells (mAECs) and vascular smooth muscle cells (VSMCs), and (**B**) quantification of their relative expression in protein extracts from *n* = 3–4 cell lots. Molecular weights (kDa) are indicated. Each data point denotes an individual experiment, and data in histograms are presented as mean ± s.e.m. One-way ANOVA with Tukey post hoc test; ****p < 0.0001, ***p < 0.001, **p < 0.01, n.s., non-significant. (**C**) Representative immunoblot analysis of CnA, CnB, and tubulin (loading control) in total aortic extracts from *n* = 10–16 Cn-Ctl and *n* = 8–10 SM-Cn^−/−^ mice, and (**D**) quantification of their relative expression in protein extracts. Molecular weights (kDa) are indicated. Each data point denotes an individual mouse, and data in histograms are presented as mean ± s.e.m. Unpaired Student *t* test; ****p < 0.0001. (**E**) Systolic blood pressure (BP), (**F**) diastolic BP, (**G**) abdominal aorta (AbAo), and (**H**) ascending aorta (AsAo) diameters in Cn-Ctl, SM-Cn^−/−^, and EC-Cn^−/−^ mice. Each data point denotes an individual mouse, and the horizontal bars denote the mean (long bar) and s.e.m. The number of mice per group is indicated in each panel. (**I**) Representative images of hematoxylin–eosin–stained AbAo and AsAo cross-sections from the indicated mice (*n* = 5 mice per genotype). Scale bar, 250 μm. Underlying data can be found in S1 Data.

To investigate the selective contribution of EC-expressed and VSMC-expressed Cn to pathological VWR, we generated tamoxifen (Tx)-inducible knockout mice conditionally lacking Cn in ECs or smooth muscle cells (SMCs). The catalytic subunit CnA is encoded by 3 genes (*Ppp3ca, Ppp3cb, and Ppp3cc*), and the regulatory subunit CnB by 2 genes (*Ppp3r1* and *Ppp3r2*). The CnB2 subunit is expressed only in the testes, whereas CnB1 is ubiquitously expressed and is indispensable for maintaining Cn activity [13]. CnA and CnB are obligate heterodimers, such that if CnB is missing, CnA becomes unstable and is degraded [14]. We therefore crossed LoxP-flanked *Ppp3r1* (*Cnb1*^f/f^) mice [15] with mice expressing Tx-inducible Cre recombinase (Cre^ERT2^) specifically in ECs (*Cdh5*-Cre^ERT2^) [16] or in SMCs (*Myh11*-Cre^ERT2^) [17], obtaining *Cnb1*^f/f^;*Cdh5*-Cre^ERT2^ mice (EC-Cn^f/f^) or *Cnb1*^f/f^;*Myh11*-Cre^ERT2^ mice (SM-Cn^f/f^), respectively. *Cnb1*^f/f^-Cre^ERT2-neg^ together with *Cnb1*^wt/wt^;*Cdh5*-Cre^ERT2^ or *Cnb1*^wt/wt^;*Myh11*-Cre^ERT2^, were used as controls (Cn-Ctl) for *Cnb1*^f/f^;*Cdh5*-Cre^ERT2^ or for *Cnb1*^f/f^;*Myh11*-Cre^ERT2^, respectively. Tx treatment induced *Cnb1* deletion specifically in ECs (EC-Cn^−/−^ mice) or SMCs (SM-Cn^−/−^ mice), as confirmed by immunoblot analysis of CnB expression in mouse aortic endothelial cells (mAECs) from EC-Cn^−/−^ mice (Fig 1A and 1B) or in aortic extracts from SM-Cn^−/−^ mice (Fig 1C and 1D). Destabilization of the catalytic subunit CnA in the absence of the regulatory subunit CnB has been demonstrated *in vivo* [14,18]. As expected, *Cnb1* deletion caused a drop in CnA protein expression (Fig 1A–1D). Of note, baseline measurements showed that Cn deficiency in ECs or SMCs did not substantially affect blood pressure (BP), aortic diameter, or aortic tissue organization (Fig 1E–1I).

**Fig 2:**
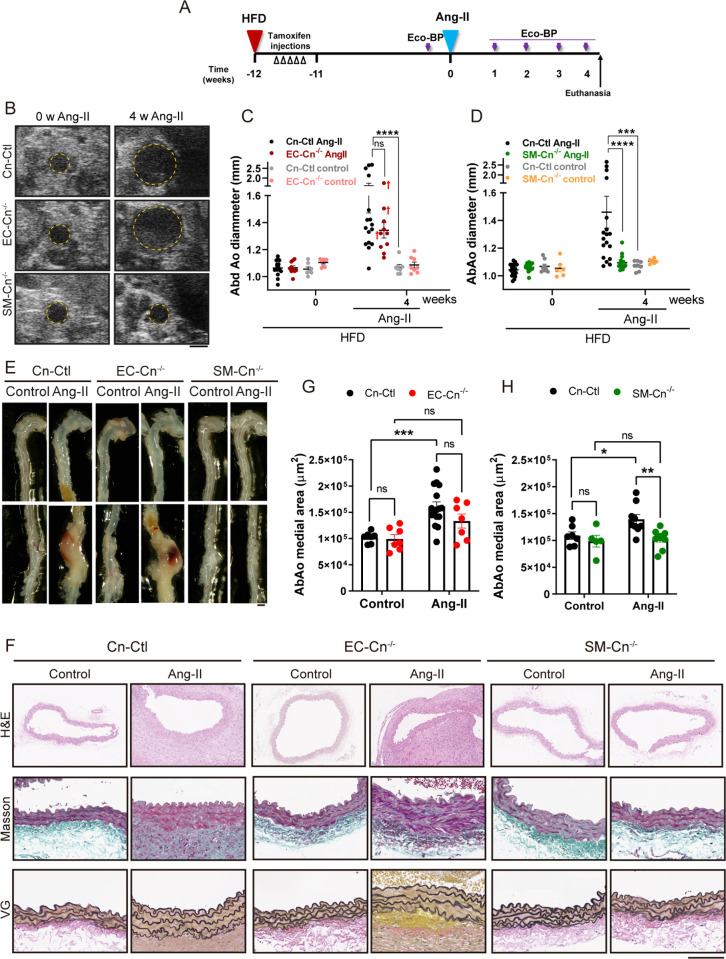
Smooth muscle cell (SMC)calcineurin (Cn) is required for angiotensin-II (Ang-II)-induced abdominal aortic aneurysm formation. **(A)** Experimental design: 10–12-week-old mice were treated with tamoxifen for five consecutive days (open arrowheads) during the first week of high-fat diet (HFD) feeding. After 12 weeks of HFD, Ang-II osmotic minipumps were implanted for 4 weeks; control mice were operated without minipump implantation. Maximum aortic diameter and BP were measured (Eco-BP) at the indicated time points (purple arrows), and mice were euthanized at the end of the experiment. (**B**) Representative ultrasound images of AbAo from mice before and after 4 weeks of Ang-II treatment. Yellow lines mark the lumen boundary. Scale bar, 1 mm. (**C, D)** AbAo diameter in (C) 7 Cn-Ctl and 8 EC-Cn^−/−^ control-treated mice and 16 Cn-Ctl and 10 EC-Cn^−/−^ Ang-II-treated, and in (D) 8 Cn-Ctl and 6 SM-Cn^−/−^ control-treated mice and 18 Cn-Ctl and 16 SM-Cn^−/−^ Ang-II-treated mice. Each data point denotes an individual mouse, and the horizontal bars denote the mean (long bar) and s.e.m. ****p < 0.0001, ***p < 0.001; repeated-measurements (RM) two-way ANOVA with Tukey’s post hoc test. Red crosses (†) indicate mice that died before completion of the experiment and exhibited a ruptured aneurysm. (**E**) Representative images of aortas from mice treated as indicated. Scale bar, 1 mm. (**F**) Representative images of hematoxylin–eosin (HE), Masson trichrome (Masson), and Van Gieson (VG) staining on AbAo sections from 4 Cn-Ctl, 3 EC-Cn^−/−^, and 4 SM-Cn^−/−^ mice. Scale bar, 100 µm. Medial area in AbAo sections from (**G**) 7 Cn-Ctl and 7 EC-Cn^−/−^ control-treated mice and 16 Cn-Ctl and 7 EC-Cn^−/−^ Ang-II-treated mice and (**H**) 7 Cn-Ctl and 5 SM-Cn^−/−^ control-treated mice and 9 Cn-Ctl and 9 SM-Cn^−/−^ Ang-II-treated mice. Each data point denotes an individual mouse, and data in histograms are presented as mean ± s.e.m. ***p < 0.001, **p < 0.01, *p < 0.05, n.s., non-significant; two-way ANOVA with Šídák post hoc test. Underlying data can be found in S1 Data. Data from a subset of untreated and Ang-II-treated Cn-Ctl mice were repeated in panels C and D and in panels G and H as indicated in S1 Data.

To assess the contributions of EC-expressed and SMC-expressed Cn to AAA, we used a mouse model [19] involving 4 weeks of Angiotensin II (Ang-II) infusion in mice fed a high-fat diet (HFD) for 12 weeks (Fig 2A). In Cn-Ctl mice, Ang-II treatment induced marked abdominal aorta (AbAo) dilation (Fig 2B–2E) without altering CnA or CnB expression in aortic tissues (S1A and S1B Fig). While Ang-II-induced AbAo dilatation was unaffected by deletion of Cn in ECs, it was abolished by Cn deletion in SMCs (Fig 2B–2E). Cn deletion in ECs or SMCs did not prevent HFD-induced obesity in these mice (S2 Fig). *Ex vivo* images and histological analysis of AbAo cross sections revealed AbAo remodeling characterized by increased wall thickness, collagen deposition, and elastic fiber disarray in Ang-II-treated Cn-Ctl and EC-Cn^−/−^ mice that was not present in the AbAo of SM-Cn^−/−^ mice (Fig 2E–2H). Furthermore, medial layer α-SMA levels were significantly higher in SM-Cn^−/−^ mice compared to Cn-Ctl and EC-Cn^−/−^ mice after Ang-II infusion (S3A and S3B Fig). In addition, markers of proliferation (Ki67) and inflammatory cell infiltration were substantially increased in the AbAo of Cn-Ctl and EC-Cn^−/−^ mice, but not in SM-Cn^−/−^ mice (S3C–S3F Fig). These findings demonstrate that SMC-expressed Cn, but not EC-expressed Cn, plays a critical role in Ang-II-induced AbAo wall remodeling and AAA formation.

### SMC Cn is required for Ang-II-induced hypertension

**Fig 3:**
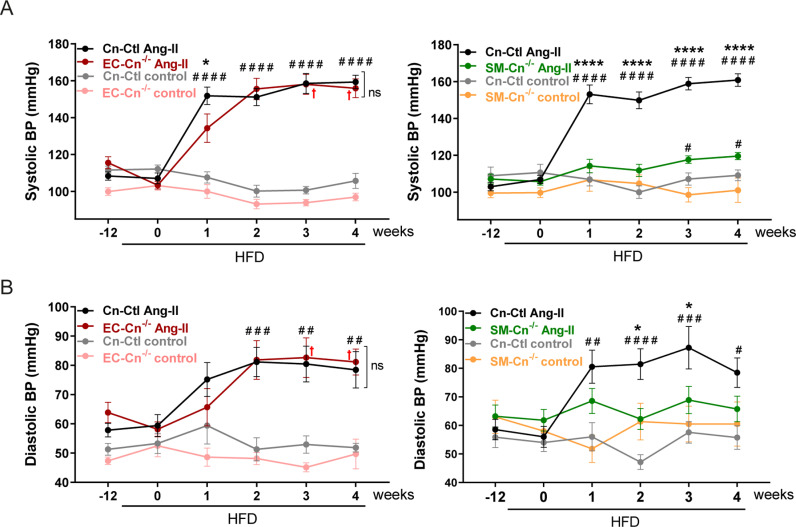
Calcineurin (Cn) deletion in smooth muscle cell (SMCs) prevents angiotensin-II (Ang-II)-driven arterial hypertension in mice fed a high-fat diet (HFD). The blood pressure (BP) was measured in the mice shown in Fig 2. (**A**) Systolic and (**B**) diastolic BP values at the indicated times in 7 Cn-Ctl and 8 EC-Cn^−/−^ control-treated mice and 16 Cn-Ctl and 10 EC-Cn^−/−^ Ang-II-treated mice (left panels) and 8 Cn-Ctl and 6 SM-Cn^−/−^ control-treated mice and 18 Cn-Ctl and 16 SM-Cn^−/−^ Ang-II-treated mice (right panels). Red crosses (†) indicate EC-Cn^−/−^ mice that died before completion of the experiment and exhibited a ruptured aneurysm. Data are presented as mean ± s.e.m. ****p < 0.0001, *p < 0.05 vs. EC-Cn^−/−^ Ang-II or vs. SM-Cn^−/−^ Ang-II, ^####^*p* < 0.0001, ^###^*p* < 0.001, ^##^*p* < 0.01, and ^#^*p* < 0.05 vs. Cn-Ctl control, and (A, right) ^#^*p* < 0.05 vs. SM-Cn^−/−^ control, n.s., non-significant; RM two-way ANOVA with Tukey’s post hoc test. Underlying data can be found in S1 Data. Data from a subset of untreated and Ang-II-treated Cn-Ctl mice were repeated in right and left panels of Figures A and B as indicated in S1 Data.

Ang-II induces vasoconstriction and hypertension in mice, and we therefore investigated whether Cn deficiency affected Ang-II-induced hypertension in the same mice used for the experiments presented in Fig 2. While Cn-Ctl and EC-Cn^−/−^ mice showed a sharp and sustained increase in systolic and diastolic BP after 1 week of treatment with Ang-II, SM-Cn^−/−^ mice remained normotensive (Fig 3). These results strongly suggest that SMC Cn is a critical mediator of Ang-II-induced hypertension.

**Fig 4:**
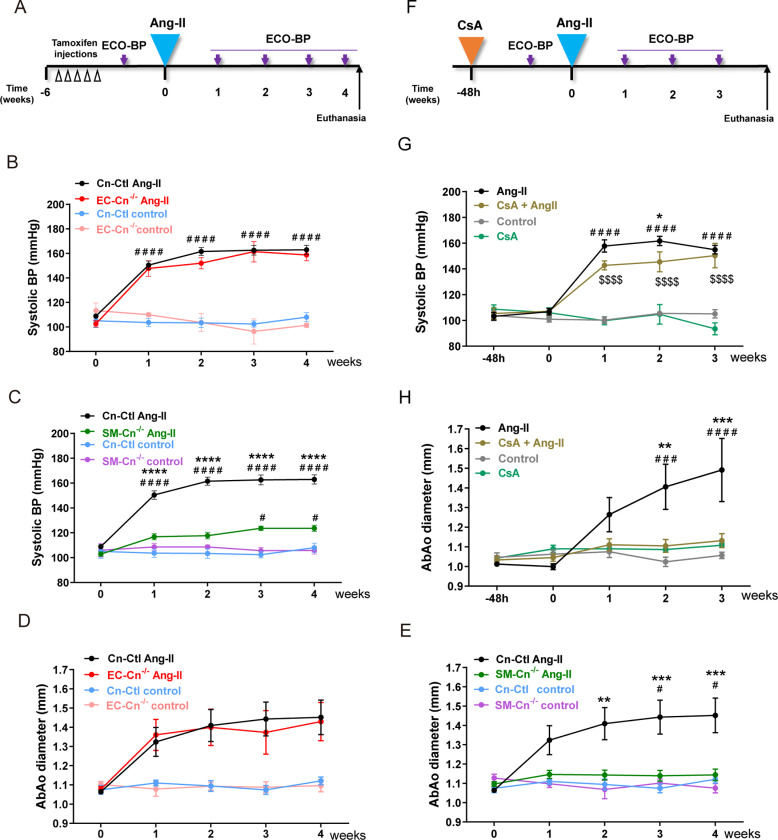
Smooth muscle cell (SMC) calcineurin (Cn) mediates angiotensin-II (Ang-II)-induced hypertension independently of its phosphatase activity. (**A**) Experimental design: 10–12-week-old mice were treated with tamoxifen for five consecutive days (open arrow heads) and, after 6 weeks, Ang-II osmotic minipumps were implanted for 4 weeks in one group of mice; control mice were operated without minipump implantation. Maximum aortic diameter and blood pressure (BP) were measured (Eco-BP) at the indicated time points (purple arrows), and mice were euthanized at the end of the experiment. (**B, C**) Systolic BP and (**D, E**) maximal abdominal aorta (AbAo) diameters at the indicated times in 5 Cn-Ctl, 3 EC-Cn^−/−^, and 5 SM-Cn^−/−^ control-treated mice and 22 Cn-Ctl, 10 EC-Cn^−/−^, and 18 SM-Cn^−/−^ Ang-II-treated mice. Data are presented as mean ± s.e.m. ****p < 0.0001, ***p < 0.001, **p < 0.01 vs. SM-Cn^−/−^ Ang-II, ^####^*p* < 0.0001 vs. Cn-Ctl control, ^#^*p* < 0.05 vs. SM-Cn^−/−^ or Cn-Ctl control; RM two-way ANOVA with Tukey’s post hoc test. (**F**) Experimental design: 10–12-week-old mice were fitted with cyclosporine A (CsA) osmotic minipumps; control mice were operated without minipump implantation. After 48 h, Ang-II minipumps were implanted in a group of CsA-treated mice (CsA + Ang-II) and in a group of CsA control mice (Ang-II). The remaining CsA control mice were operated without minipump implantation (Control). Maximal aortic diameter and BP were measured (Eco-BP) at the indicated time points (purple arrows), and mice were euthanized at the end of the experiment. (**G**) Systolic BP and (**H**) maximal AbAo diameters at the indicated times in 8 Control-, 10 Ang-II-, 4 CsA-, and 8 CsA + Ang-II-treated mice. Data are presented as mean ± s.e.m. ***p < 0.001, **p < 0.01, *p < 0.05 vs. CsA + Ang-II, ^####^*p* < 0.0001, ^###^*p* < 0.001 vs. control, ^$$$$^*p* < 0.0001 vs. CsA; RM two-way ANOVA with Tukey’s post hoc test. Underlying data can be found in S1 Data. Data from untreated and Ang-II-treated Cn-Ctl mice were repeated in panels B and C, and in panels D and E as indicated S1 Data.

In rabbits, a high-cholesterol diet alters Ang-II signaling in the aorta [20]. Therefore, to exclude possible secondary effects of HFD feeding on BP regulation by SMC Cn, we investigated the contribution of vascular Cn to Ang-II-induced hypertension in mice fed a chow diet. Infusion of Ang-II in Tx-treated mice (Fig 4A) sharply increased BP in Cn-Ctl and EC-Cn^−/−^ mice from the first week of treatment but failed to substantially raise BP in SM-Cn^−/−^ mice (Figs 4B, 4C, and S4). Similarly, Ang-II induced AbAo dilatation in Cn-Ctl and EC-Cn^−/−^ mice from the first week of treatment but did not have this effect in SM-Cn^−/−^ mice (Fig 4D and 4E). These results support a key role for SMC Cn in Ang-II-induced hypertension and AbAo dilatation.

To investigate whether Cn deletion in SMCs also conferred resistance to hypertension induced by other factors, we treated mice with norepinephrine (NE) or the nitric oxide synthase inhibitor L-NAME (S5A Fig). NE and L-NAME both markedly increased BP in Cn-Ctl mice relative to similarly treated SM-Cn^−/−^ mice and untreated controls (S5B Fig). These results indicate that SMC-expressed Cn is an essential regulator of BP in response to diverse hypertensive stimuli.

### Ang-II-induced hypertension does not require Cn phosphatase activity

A major role for Cn in BP regulation was unexpected because inhibition of Cn activity does not prevent Ang-II-induced hypertension in mice [21] and because long-term treatment of patients with Cn inhibitors causes hypertension, likely as a consequence of nephrotoxicity [22]. A number of factors could account for this discrepancy, including different contributions of Cn to BP regulation in humans and mice, off-target effects of Cn inhibitors, or a distinct effect of pharmacological inhibition versus protein deletion. Pretreatment of mice with the Cn inhibitor CsA did not protect against Ang-II-induced hypertension in WT mice (Fig 4F and 4G); in contrast, CsA pretreatment did block Ang-II-induced AbAo dilatation in these mice (Fig 4H). This result confirms effective phosphatase inhibition and is consistent with a previous report showing that CsA prevents AbAo dilatation and AAA [7]. Demonstrating efficient systemic inhibition of Cn phosphatase activity by CsA, NFATc3, a known Cn substrate, was hyperphosphorylated in the thymus of CsA-treated mice (S6A Fig). Also consistent with previous reports [21], CsA pretreatment significantly reduced Ang-II-induced cardiac hypertrophy in these mice (S6B and S6C Fig). Together, these data indicate that CsA does not prevent Ang-II-induced hypertension and thus suggest that the contribution of Cn to Ang-II-induced hypertension is unrelated to its phosphatase activity.

**Fig 5:**
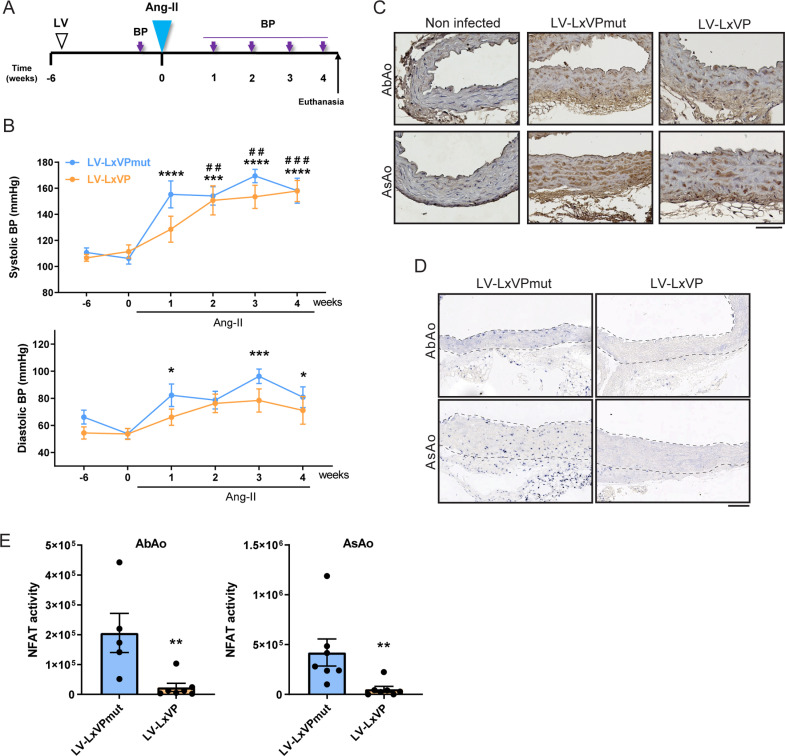
Calcineurin (Cn) inhibition by LxVP lentiviral transduction impairs NFAT activation *in vivo* without preventing angiotesin-II (Ang-II)-induced hypertension. (**A**) Experimental design: 10–12-week-old mice were inoculated with LxVPmut- or LxVP-encoding lentivirus (LV) and, after 6 weeks, Ang-II osmotic minipumps were implanted for 4 weeks. BP was measured at the indicated time points (purple arrows), and mice were euthanized at the end of the experiment. (**B**) Systolic and diastolic BP in mice transduced with LV-LxVPmut (*n* = 7) and LV-LxVP (*n* = 7), shown as mean ± s.e.m. ****p < 0.0001, ***p < 0.001, **p < 0.01, *p < 0.05 and ^###^*p* < 0.001, ^##^*p* < 0.01 vs. beginning of treatment (0 weeks) for LV-LxVPmut- and LV-LxVP-transduced mice, respectively; RM two-way ANOVA with Tukey’s post hoc test. (**C**) Representative images of GFP immunostaining in abdominal aorta (AbAo) and ascending aorta (AsAo) cross-sections from the indicated mice. (**D**) Representative images of southwestern histochemistry with an NFAT probe of AbAo and AsAo cross-sections from the indicated mice and (**E**) quantification of the relative staining (NFAT activity) in the AbAo (left panel) and the AsAo (right panel). Dashed lines indicate the medial layer. Only mice with positive aortic GFP staining and consistent NFAT activity were included. Each data point denotes an individual mouse, and data in histograms are presented as mean ± s.e.m. Mann–Whitney test **p < 0.01. Scale bars, 100 μm. Underlying data can be found in S1 Data.

To confirm the non-involvement of Cn phosphatase activity in Ang-II-induced hypertension, we inhibited Cn activity *in vivo* by inoculating WT mice with a lentivirus (LV) encoding the LxVP peptide fused to GFP (LV-LxVP). Like CsA, the LxVP peptide blocks the binding of Cn to its substrates, inhibiting its activity [23]. Six weeks after LV inoculation, Ang-II was administered to mice inoculated with LV-LxVP or with LV encoding an inactive mutant version (LV-LxVPmut), and BP was determined (Fig 5A). Ang-II markedly increased systolic and diastolic BP in mice transduced with LV-LxVPmut or LV-LxVP, with no substantial difference between treatment groups (Fig 5B). Efficient transduction of all aortic layers was confirmed by GFP immunostaining of aortic sections (Fig 5C). To assess whether LV-LxVP efficiently blocked Cn/NFAT activation by Ang-II in these mice, we analyzed the subcellular localization and DNA-binding activity of NFAT proteins. NFATs are dephosphorylated by Cn and translocate to the nucleus in active form to bind target gene promoters. *In situ* southwestern histochemistry detected abundant active NFAT in cell nuclei in medial AbAo and ascending aorta (AsAo) sections from Ang-II-treated mice expressing LV-LxVPmut, but a nuclear NFAT signal was barely detected in sections from LV-LxVP transduced animals (Fig 5D and 5E). These data further support the idea that SMC Cn mediates Ang-II-induced hypertension independently of its phosphatase activity.

### SMC Cn is required for both early onset and long-term sustainability of Ang-II-induced hypertension

**Fig 6:**
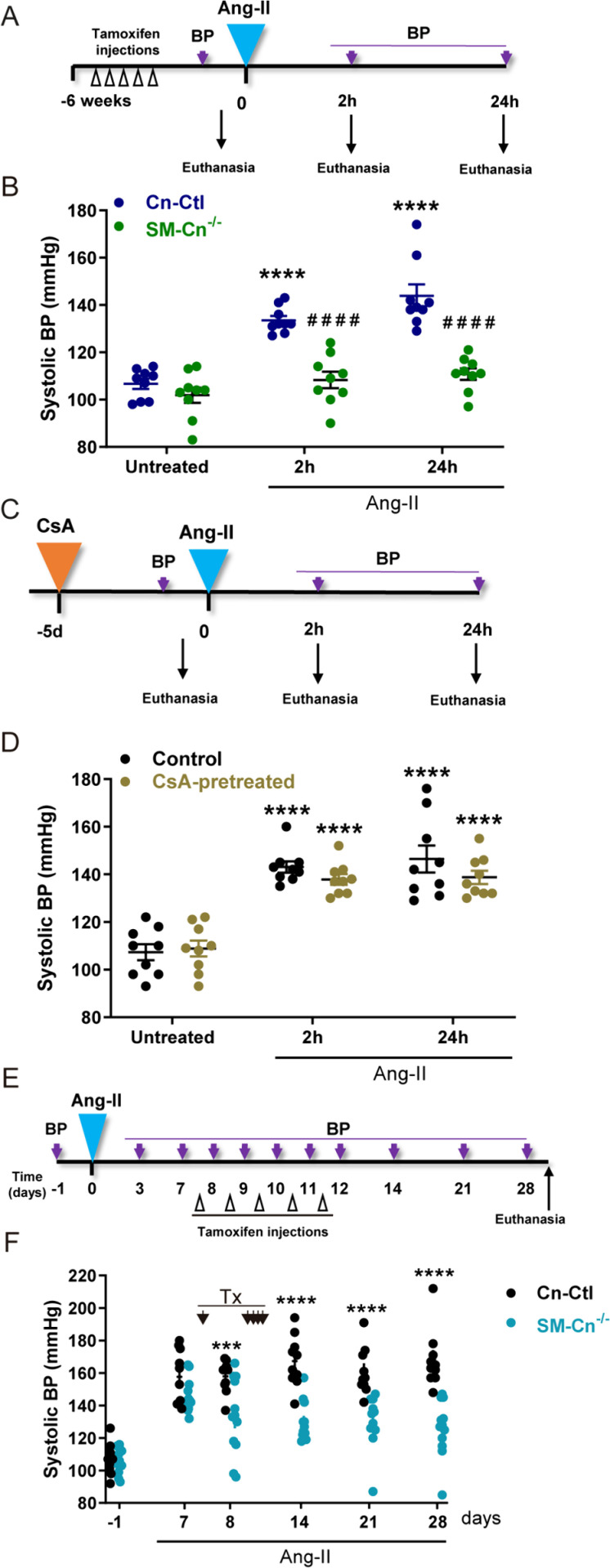
Smooth muscle cell (SMC) calcineurin (Cn) is required for both the onset and the long-term sustainability of angiotensin-II (Ang-II)-induced hypertension. (**A**) Experimental design: 10-12-week-old mice were treated with tamoxifen for five consecutive days (open arrow heads) and, after 6 weeks, Ang-II osmotic minipumps were implanted for 2 and 24 h. BP was measured at the indicated time points (purple arrows), and mice were euthanized and analyzed when indicated. (**B**) Systolic BP values in the indicated mice (*n* = 9 mice per group and time point). Each data point denotes an individual mouse, and the horizontal bars denote the mean (long bar) and s.e.m. ****p < 0.0001 vs. baseline; RM two-way ANOVA with Tukey post hoc test. ^####^*p* < 0.0001 vs. 2 or 24 h Ang-II Cn-Ctl; RM two-way ANOVA with the Šídák post hoc test. (**C**) Experimental design: 10–12-week-old mice were fitted with CsA osmotic minipumps (CsA-pretreated); control mice were operated without minipump implantation (Control). After 5 days, Ang-II minipumps were implanted in CsA-pretreated and Control mice for 2 and 24 h. BP was measured at the indicated time points (purple arrows), and mice were euthanized and analyzed when indicated. (**D**) Systolic BP in the indicated mice (*n* = 9 mice per group and time point). Each data point denotes an individual mouse, and the horizontal bars denote the mean (long bar) and s.e.m. ****p < 0.0001 vs. baseline; RM two-way ANOVA with Tukey’s post hoc test. (**E**) Experimental design: 10–12-week-old mice were fitted with Ang-II osmotic minipumps for 4 weeks. After 7 days of Ang-II infusion, tamoxifen was administered for five consecutive days (open arrow heads). BP was measured at the indicated time points (purple arrows), and mice were euthanized at the end of the experiment. (**F**) Systolic BP measurements at the indicated times in 10 Cn-Ctl and 12 SM-Cn^−/−^ mice. Each data point denotes an individual mouse, and the horizontal bars denote the mean (long bar) and s.e.m. ****p < 0.0001, ***p < 0.001 vs. Cn-Ctl Ang-II; RM two-way ANOVA with Šídák post hoc test. Tx indicates tamoxifen administration time points. Underlying data can be found in S1 Data.

To investigate whether smooth muscle Cn was required only for the long-term maintenance of Ang-II-induced hypertension or also for its onset, we measured BP in Cn-Ctl and SM-Cn^−/−^ mice early after Ang-II infusion (Fig 6A). A robust increase in BP was observed in Cn-Ctl mice as early as 2 h after Ang-II infusion and was maintained after 24 h, whereas SM-Cn^−/−^ mice remained normotensive (Figs 6B and S7A). In parallel studies, WT mice were treated with CsA for 5 days before Ang-II infusion (Fig 6C). Consistent with the results of the long-term experiment with CsA (Fig 4F and 4G), CsA pre-treatment failed to prevent Ang-II-induced hypertension after 2 or 24 h (Figs 6D and S7B). Efficient inhibition of Cn phosphatase activity in CsA-treated mice was confirmed by the NFAT phosphorylation status in the thymus and the inhibition of aortic *Rcan1-4* mRNA expression (S7C and S7D Fig). These results strongly suggest that Ang-II activates molecular mechanisms critical for the early increase in BP that require Cn expression in SMCs, but not its phosphatase activity.

To determine whether the presence of Cn is required for long-term, sustained hypertension, we triggered Cn deletion in SMCs 7 days after inducing hypertension with Ang-II (Fig 6E). Treatment with Ang-II for 7 days elicited a similar degree of hypertension in Cn-Ctl and SM-Cn^f/f^ mice (Fig 6F). At this point, all mice were treated with Tx to elicit smooth muscle deletion of Cn in the SM-Cn^f/f^ mice, generating SM-Cn^−/−^ mice. BP decreased in SM-Cn^−/−^ mice as early as 1 day after the first Tx administration and continued to decline until the end of the experiment; in contrast, Cn-Ctl mice remained hypertensive until the end of the experiment (Fig 6F). These data strongly suggest that smooth muscle Cn is actively involved in the onset and maintenance of Ang-II-induced hypertension, identifying smooth muscle Cn as a potential target for the treatment of hypertension.

### SMC Cn orchestrates the Ang-II-induced transcriptional program involved in arterial contractility and hypertension

**Fig 7:**
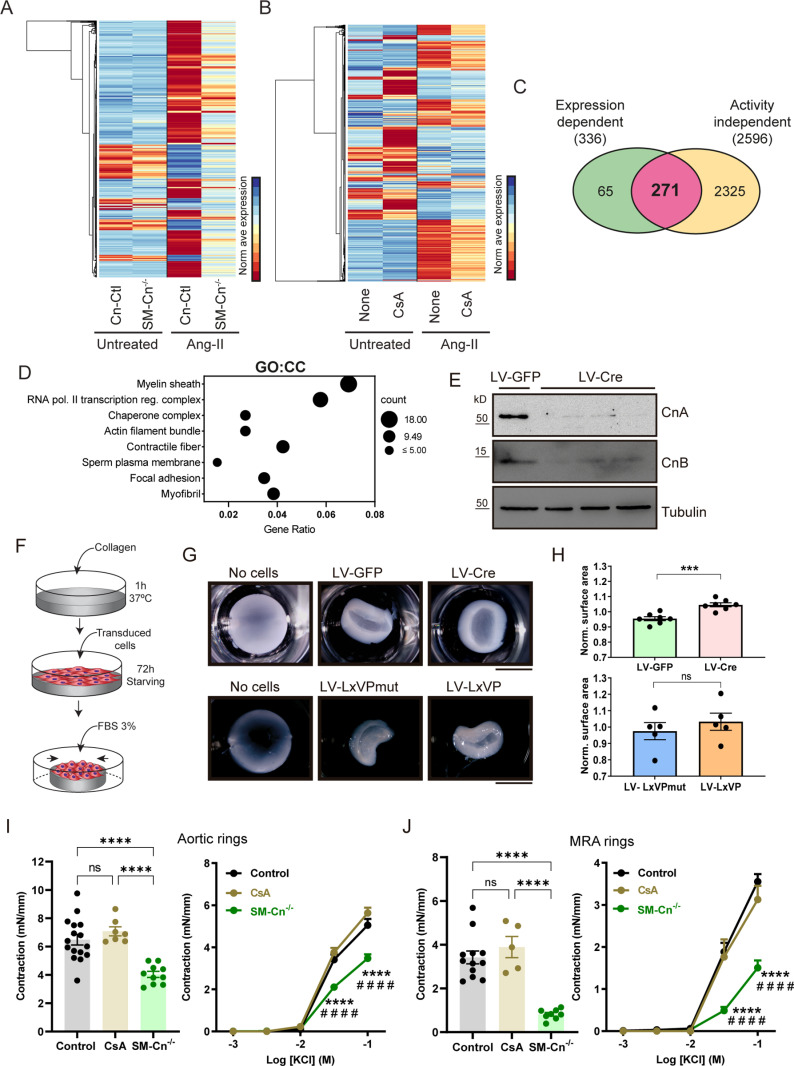
Smooth muscle cell (SMC) calcineurin (Cn) orchestrates an angiontensin-II (Ang-II)-induced transcriptional program related to arterial contractility. (**A, B**) Heatmaps of the normalized average expression of differentially expressed genes (DEGs) regulated by angiotensin-II (Ang-II) relative to the untreated conditions in each independent RNAseq dataset. (**C**) Venn diagram comparing the number of Cn expression-dependent DEGs (336) and Cn activity-independent DEGs (2,596), and showing the number of shared DEGs whose regulation by Ang-II requires Cn expression but not its activity (271). (**D**) Most significantly enriched cellular components (CC) of the 271 Cn expression-dependent DEGs, obtained by g:Profiler. (**E**) Representative immunoblot analysis (*n* = 7 individual experiments) of CnA, CnB, and tubulin (loading control) in protein extracts from primary VSMCs transduced with GFP- or Cre-encoding lentivirus (LV-GFP- or LV-Cre, respectively). Molecular weights (kDa) are indicated. (**F**) Experimental design: Collagen gels were polymerized for 1 h at 37 °C, and VSMCs (8 × 10^4^) were seeded on each gel, allowed to adhere for 5 h, and serum-starved for 72 h. Gels were then gently detached from the plates and incubated in DMEM supplemented with 3% FBS for 24—48 h at 37 °C. (**G**) Representative images and (**H**) normalized surface area of fixed collagen gels under the indicated conditions. Each data point denotes the mean of one experiment, and data in histograms are presented as mean ± s.e.m. (*n* = 7 independent experiments with LV-GFP and LV-Cre; *n* = 5 independent experiments with lentivirus encoding the LxVP peptide or its inactive form LxVPmut). ***p < 0.001; n.s., non-significant; unpaired Student *t* test. Scale bars, 5 mm. Quantification of tension in (**I**) aortic rings and (**J**) mesenteric resistance artery (MRA) rings from control (Cn-Ctl or WT), SM-Cn^−/−^, or CsA-treated WT mice after stimulation for 30 min with 80 mM KCl (left panels) and the indicated KCl concentrations (right panels). Left panels: each data point denotes an individual mouse, and data in histograms are presented as mean ± s.e.m. ****p < 0.0001; one-way ANOVA with Tukey post hoc test. Right panels: data are shown as mean ± s.e.m. ****p < 0.0001 vs. control, ^####^*p* < 0.0001 vs. CsA; two-way ANOVA with Tukey post hoc test. Aortic rings experiments including samples from 16 control, 10 SM-Cn^−/−^, and 7 CsA-treated mice, while MRA rings were anlyzed from 12 control, 8 SM-Cn^−/−^, and 5 CsA-treated mice. Underlying data can be found in S1 Data.

To gain insight into the Cn-mediated molecular mechanisms underlying the early onset of hypertension, we performed a transcriptomic analysis to identify genes whose regulation by Ang-II in the aorta requires Cn expression in SMCs. To obtain an unbiased overview of gene expression changes in response to Ang-II, we selected all differentially expressed genes (DEGs) upon Ang-II treatment in each genotype. Specifically, we compared untreated versus Ang-II-treated Cn-Ctl mice and untreated versus Ang-II-treated SM-Cn^−/−^ mice, selecting protein-coding genes with a fold change (FC) > ±2. This set of genes was considered sensitive to Ang-II treatment. Heatmap analysis of these DEGs revealed that smooth muscle Cn is essential for the differential expression of most Ang-II-regulated genes in the aorta (Fig 7A). When analyzing DEGs independently of the FC threshold, we identified 800 DEGs in the aortas of Cn-Ctl mice treated for 2 h with Ang-II versus untreated mice; of these DEGs, almost 90% (722 genes) showed no evidence of Ang-II regulation in the aortas of SM-Cn^−/−^ mice (Figs 7A and S8A and S1 Table). To refine the list of candidate mediators involved in Ang-II-induced hypertension, we focused on a subset of 336 DEGs that were also differentially expressed between Ang-II-treated Cn-Ctl and SM-Cn^−/−^ mice (S8B Fig and S2 Table).

Since CsA pretreatment did not prevent Ang-II-induced hypertension, we reasoned that genes whose induction by Ang-II was sensitive to CsA would be unlikely to mediate hypertension. We therefore performed an additional transcriptomic analysis to identify genes whose regulation by Ang-II was unaffected by CsA. The performance of this study was very high and identified 2,653 DEGs in the aortas of mice treated with Ang-II for 2 h (S8C Fig and S3 Table). To obtain an unbiased overview of gene expression in this context, we selected protein-coding DEGs with an FC > ±2 from comparisons between untreated and Ang-II-treated WT mice, and between untreated and AngII-treated CsA-pretreated mice. Heatmap analysis of these DEGs showed a marked effect of Ang-II treatment on the aortic transcriptome and a subtle effect of CsA pretreatment on gene expression regulation by Ang-II (Fig 7B), with 2,596 of the Ang-II-regulated genes (almost 98%) insensitive to CsA pretreatment (S8C Fig). Comparison of these genes with the 336 Cn-dependent DEGs identified a group of 271 genes whose regulation by Ang-II required Cn expression but not its phosphatase activity (Fig 7C). Functional annotation clustering of these genes revealed enrichment in cluster terms strongly associated with cellular components involved in cell contractility regulation, such as “actin filament bundle”, “contractile fiber”, “focal adhesion”, and “myofibril” (Fig 7D).

VSMC contractility has a direct effect on BP regulation [3], and actomyosin cytoskeleton dynamics is critical for VSMC contractility regulation [24]. To investigate the influence of Cn on VSMC contractile capacity, we cultured primary Cn^f/f^ VSMCs transduced with GFP-encoding or Cre-encoding lentivirus (LV-GFP or LV-Cre, respectively) on collagen disks. In this assay, contractile capacity is determined by measuring the collagen disk surface area, which decreases as the attached cells contract in response to 3% fetal bovine serum (FBS) (Figs 7F and S9) [25,26]. Transduction with Cre-encoding LV efficiently deleted Cn expression (Fig 7E) and substantially impaired gel contraction relative to cells transduced with LV-GFP (Fig 7G and 7H). In contrast, transduction of WT VSMCs with an LxVP-encoding lentivirus (LV-LxVP) failed to prevent gel contraction (Fig 7G and 7H). These results suggest that Cn regulates VSMC contractility through a mechanism unrelated to its phosphatase activity. To investigate the contribution of SMC Cn to vessel contractility regulation in a more physiological setting, we measured the KCl-induced contraction of vehicle- or CsA-pretreated aortic and mesenteric resistance artery (MRA) rings from WT, Cn-Ctl, and SM-Cn^−/−^ mice. Cn deficiency, but not inhibition of its phosphatase activity with CsA, decreased aortic and MRA contractility in response to saturating (Fig 7I and 7J, left panels) or increasing KCl concentrations (Fig 7I and 7J, right panels), providing further support for the idea that Cn expression in SMCs is critical for arterial contractility regulation independently of Cn phosphatase activity.

**Fig 8:**
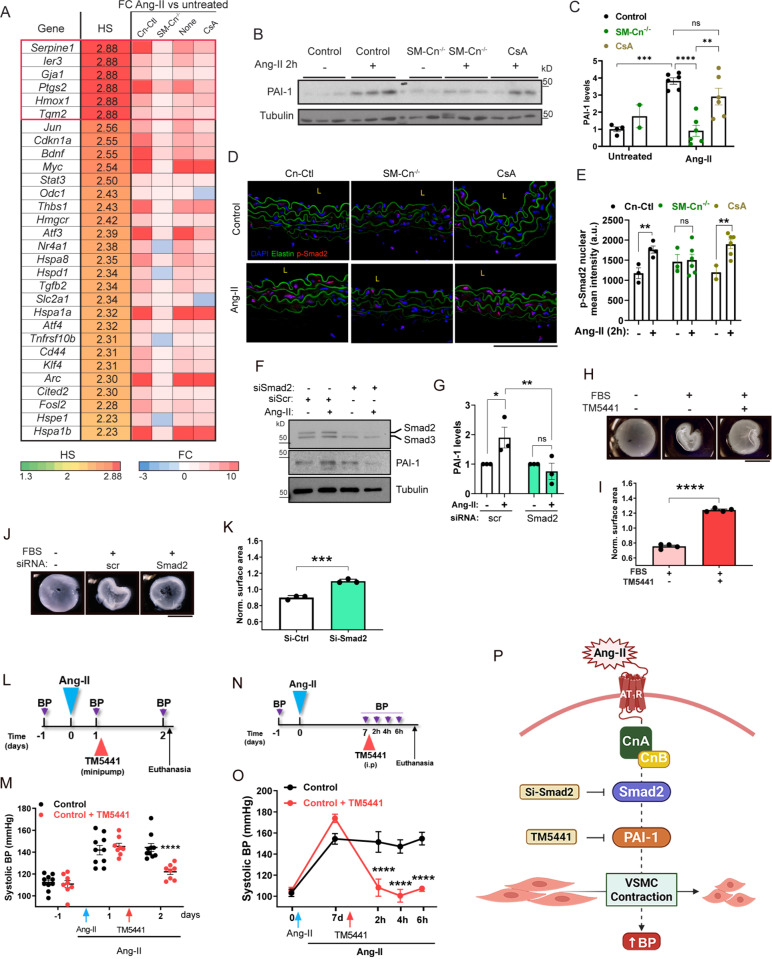
Plasminogen activator inhibitor type-1 (PAI-1) mediates vascular smooth muscle cell (VSMC) contractility and angiotensin-II (Ang-II)-induced hypertension. (**A**) List of the top 30 genes with the highest hypertension score (HS), including their gene symbol, HS, and expression fold change (FC) after 2 h of Ang-II treatment relative to untreated mice. (**B**) Representative immunoblot analysis of PAI-1 and tubulin (loading control) and (**C**) quantification of their relative expression in protein extracts from untreated control (*n* = 4) and SM-Cn^−/−^ (*n* = 2) mice and from Ang-II-treated control (*n* = 6), SM-Cn^−/−^ (*n* = 6), and CsA-pretreated (*n* = 6) mice. Molecular weights (kDa) are indicated. One-way ANOVA with Tukey post hoc test; ****p < 0.0001, ***p < 0.001, **p < 0.01, n.s., non-significant. (**D**) Representative images of phospho-Smad2 immunostained AbAo cross-sections from the indicated group of mice. L, lumen. Scale bar, 100 μm. (**E**) Quantification of the mean intensity of the phospho-Smad2 nuclear signal in the same cross-sections. Each data point denotes an individual mouse, with values representing the average quantification of at least two independent images per mouse. Data in histograms are presented as mean ± s.e.m. **p < 0.01; unpaired Student *t* test. (**F**) Representative immunoblot analysis of Smad2/3, PAI-1, and tubulin (loading control) and (**G**) quantification of their relative expression in protein extracts from the indicated groups (*n* = 3 independent experiments). Molecular weights (kDa) are indicated. Data are presented as mean ± s.e.m. *p < 0.05, **p < 0.01; two-way ANOVA with Šídák post hoc test. (**H–K**) Representative images (H, J) and normalized surface area (I, K) of fixed collagen gels under the indicated conditions. Each data point denotes the mean of each experiment, and data in histograms are presented as mean ± s.e.m. (*n* = 3−4 independent experiments with 3% FBS and 50 µM TM5441 (H)). ***p < 0.001, ****p < 0,0001; unpaired Student *t* test. Scale bars, 5 mm. (**L**) Experimental design: 10–12-week-old mice were fitted with Ang-II osmotic minipumps (Ang-II) 24 h before implantating TM5441 minipumps in a group of Ang-II-pretreated mice (Ang-II + TM5441). BP was measured at the indicated time points (purple arrows), and mice were euthanized at the end of the experiment. (**M**) Systolic BP at the indicated times in 10 Ang-II-treated mice (Ang-II) and 8 mice treated with Ang-II + TM5441. Data are presented as mean ± s.e.m. ****p < 0.001, vs. Control; RM two-way ANOVA with Šídák post hoc test. (**N**) Experimental design: 10–12-week-old mice were fitted with Ang-II osmotic minipumps (Ang-II) 7 days before intraperitoneally injecting TM5441 or vehicle to a group of Ang-II-pretreated mice. BP was measured at the indicated time points (purple arrows), and mice were euthanized at the end of the experiment. (**O**) Systolic BP at the indicated times in 5 Ang-II + vehicle (Control) and 6 mice treated with Ang-II + TM5441 (Control + TM5441). Data are presented as mean ± s.e.m. ****p < 0.001, vs. Control; RM two-way ANOVA with Šídák post hoc test. (**P**) Proposed mechanism: a structural role of SMC Cn mediates the induction of PAI-1 by Ang-II via Smad2 activation, subsequently leading to VSMC contraction and the increase of blood pressure (BP). Underlying data can be found in S1 Data.

To investigate the potential association of the group of 271 genes with hypertension, we performed an analysis using the Harmonizome dataset collection (http://amp.pharm.mssm.edu/Harmonizome). This analysis provided a hypertension score (HS) for >70% of these genes (S4 Table), suggesting a likely association with hypertension and supporting the validity of our transcriptomics approach as a way to identify genes encoding mediators of Ang-II-induced hypertension. Graphical representation of the FC in expression induced by Ang-II for the genes with the highest HS revealed a markedly different regulation in SM-Cn^−/−^ mice relative to Cn-Ctl, WT, and CsA-pretreated mice (Fig 8A). We further analyzed the expression of three genes with the highest HS—*Serpine1, Ier3,* and *Gja1*—in aortas from individual mice using reverse transcription quantitative real-time polymerase chain reaction (RT-qPCR). After 2 h of Ang-II exposure, the expression of these genes was markedly inhibited in Cn-deficient mice but not in mice treated with CsA (S10 Fig), identifying them as candidate mediators of Ang-II-induced hypertension.

Consistent with the mRNA data, the expression of PAI-1, the protein encoded by *Serpine1*, was increased in aortas of mice treated with Ang-II for 2 h. This increase did not occur in SM-Cn^−/−^ mice and was not prevented by the treatment with CsA (Fig 8B and 8C). Multiple transcription factors can induce *Serpine1* expression, including p53, AP1, NF-kB, CREB1, and SMAD2/3 [27–30]. Notably, SMAD2/3 activation occurs not only via TGF-β, but also through TGF-β-independent mechanisms triggered by Ang-II [31,32]. To explore the role of these factors in Cn-mediated induction of *Serpine1* by AngII, we assessed Smad2 phosphorylation at Ser465/467, a marker of its activation. Smad2 phosphorylation was markedly increased after 2 h of Ang-II infusion in aortas from Cn-Ctl and CsA-treated mice but was not observed in aortas from SM-Cn^−/−^ mice (Figs 8D, 8E, S11A and S11B). These findings indicate that Smad2 activation by Ang-II is mediated by a phosphatase-independent activity of Cn.

To further evaluate the role of Smad2 in PAI-1 induction by Ang-II, we knocked down *Smad2* in VSMCs. *Smad2* silencing substantially reduced PAI-1 induction by Ang-II in VSMCs (Figs 8F, 8G and S11C), strongly suggesting that a structural role of Cn mediates *Serpine1* induction through Smad2 activation.

### PAI-1 is a key regulator of VSMC contractility and hypertension

Given the identification of PAI-1 as a major candidate mediator of Ang-II-induced hypertension, we investigated its potential role in regulating contractility and Ang-II-induced hypertension. Pre-treatment of VSMCs with the PAI-1 inhibitor TM5441 nearly abolished their capacity to contract collagen gels (Fig 8H and 8I), a result that was consistently replicated when Smad2 was silenced (Fig 8J and 8K).

In an *in vivo* approach, when mice were treated with PAI-1 inhibitor 24 h after the induction of hypertension with Ang-II (Fig 8L), there was a marked reduction in BP 24 h after the inhibitor administration (Fig 8M). Additionally, in a complementary experimental setup, acute intraperitoneal administration of TM5441 fully reversed hypertension induced by 7 days of Ang-II treatment (Fig 8N and 8O). These findings suggest that PAI-1 inhibition may rapidly restore normotension in hypertensive patients.

Taken together, our results emphasize the link between BP regulation and VSMC contractility, demonstrating that PAI-1 is a critical mediator of VSMC contractility and Ang-II-induced hypertension. Its expression is induced by Ang-II through Smad2 activation, which is dependent on a structural role of Cn in SMCs that does not require its phosphatase activity (Fig 8P).

## Discussion

The results of this study reveal that the phosphatase activity of SMC Cn is critical for Ang-II-induced abdominal aorta dilatation, whereas, unexpectedly, Cn drives hypertension independently of its phosphatase activity. Through this non-enzymatic function, SMC Cn emerges as a master regulator of a gene expression program that leads to hypertension. Almost 90% of the genes regulated by the vasopressor Ang-II during hypertension onset require Cn expression in SMCs, but only a small fraction of these genes require Cn phosphatase activity for this regulation. Together, our findings suggest a critical structural role for Cn in BP and gene expression regulation.

Cn was identified in previous studies as a mediator of Ang-II-induced AAA and vascular remodeling [7,12,33], yet the aortic cell types responsible for this role remained unknown. Our current findings demonstrate that Ang-II-induced AAA formation in hyperlipidemic mice requires Cn expression in SMCs, but not in ECs. Our previous research indicated that AAA formation requires the phosphatase activity of Cn [7]. With our new findings, we now show that, while the development of hypertension also requires the presence of Cn in SMCs, it does not require Cn enzymatic activity.

Although we confirmed the absence of Cn in both SMC- and EC-specific KO mice by immunoblotting, a limitation of this study is the lack of immunofluorescence validation or direct assessment of Cn/NFAT activity loss in specific vascular compartments. However, we assessed this by analyzing the expression of *Rcan1-4*, a well-characterized NFAT target gene responsive to CsA treatment [7]. Its induction by Ang-II was significantly blunted in aortas of SM-Cn ⁻ ^/^ ⁻ mice (S7D Fig), indicating reduced NFAT activity and providing functional evidence of Cn inactivation in our models.

Hypertension is a complex and multifactorial disease and, despite its high prevalence, the basis of its pathogenesis is not fully understood, creating problems in its clinical management and therapy [2]. The involvement of VSMCs in hypertension has been demonstrated in several studies [34–37]. Our present results show that specific deletion of Cn in SMCs, but not in ECs, prevents Ang-II-induced increases in systolic and diastolic BP. Moreover, Cn deletion in SMCs also inhibited hypertension induced by the vasopressors NE and L-NAME. Since SMC Cn deletion 1 week after inducing hypertension with Ang-II substantially decreased BP, our results point to an essential role for SMC Cn not only in the onset but also in the maintenance of hypertension. A recent report suggested that the catalytic subunit CnAβ is required for sustained Ang-II-induced hypertension but not for its induction, since constitutive CnAβ deletion did not prevent Ang-II-induced hypertension during the first 3 weeks of treatment but did cause a decrease in systolic BP 1 week later [33]. Since *Cnb1* deletion in SMCs leads to destabilization of both Cn catalytic subunits expressed in these cells (CnAα and CnAβ) and blocks Ang-II-induced hypertension, we propose that while CnAα mediates the induction of hypertension by Ang-II, CnAβmay be required for its maintenance.

Inhibition of Cn phosphatase activity with the immunosuppressor CsA prevented Ang-II-induced aortic dilatation but did not prevent Ang-II-induced hypertension. This discrepancy between pharmacological inhibition and genetic ablation may stem from previously reported hypertensive effects of long-term CsA treatment [38–40] or from off-target effects of CsA on apoptotic pathways [41], cyclophilin D [42], or cyclophilin B [43]. These off-target effects might also explain the differences in gene regulation observed between untreated CsA-pretreated and untreated WT mice (Fig 7B). However, we detected no BP increase in mice treated with CsA alone. It should be noted that prior studies used higher CsA doses (20–30 mg/kg/d, versus 5 mg/kg/d in this study) and different administration routes [38–40]. These results suggest that SMC Cn mediates Ang-II-induced hypertension through a mechanism independent of its phosphatase activity, an idea supported by our finding that LVs encoding the Cn inhibitory peptide LxVP also failed to impede Ang-II-induced hypertension despite efficiently blocking activation of the Cn substrate NFAT.

Catalysis-independent roles have already been described for some enzymes; for example, short-term presynaptic plasticity requires the presence of αCamKII but not its kinase activity [44]. Although Cn function has broadly been attributed to its phosphatase activity, a phosphatase-independent role has been proposed in cardiac fibrosis [21], and demonstrated in postsynaptic density distribution [45] and assembly of a functional TCR signaling complex [46]. Moreover, Cn interaction with its substrate NFAT leads not only to its dephosphorylation-mediated activation but also to its retention in the nucleus by masking the nuclear export signal [47]. New Cn interactors and Cn-binding sites distinct from the canonical LxVP and PIxIT sites have been identified [48] and some of the identified Cn interactors can serve as Cn scaffolds or regulate Cn enzymatic activity or its subcellular distribution [49,50], suggesting the possibility that Cn might be part of a macromolecular signaling complex. In this line, our results suggest that some Cn interactors might mediate BP regulation independently of Cn phosphatase activity.

Although BP is largely determined by the resistance arteries, large arteries also contribute by regulating pulse wave pressure during the cardiac cycle, and changes in aortic stiffness or compliance are strongly associated with hypertension in humans [51]. There is increasing evidence that large artery damage has a deleterious impact on the microcirculation that affects hypertension; however, the underlying molecular mechanisms are poorly understood [4]. Since we detected hypertension as early as 2 h after Ang-II infusion in WT and Cn-Ctl mice, we reasoned that a transcriptomic analysis of the aorta performed at this time could identify genes mediating hypertension onset. The regulation of nearly 90% of the genes affected by Ang-II in the aorta required Cn expression in SMCs, indicating that among the numerous signaling pathways activated by Ang-II, those mediated by Cn are critical, at least in the aorta, for early signaling events. Consistent with our previous results showing that just 11 of >1,500 Ang-II-regulated genes in cultured VSMCs are sensitive to Cn inhibition by CsA [7], only 2% of the transcriptome regulated by Ang-II in the aorta required Cn phosphatase activity. These results further support a structural, phosphatase-activity-independent role for Cn in gene expression regulation.

To identify candidate mediators of hypertension, we focused on genes whose regulation by Ang-II was dependent on Cn expression but not on its phosphatase activity. Functional annotation clustering analysis of these genes revealed that Cn orchestrates the induction of a gene expression program enriched in cellular components strongly associated with VSMC contractility regulation. Moreover, Cn deletion in cultured VSMCs, but not inhibition of its phosphatase activity, impaired the contractile capacity of these cells. Accordingly, Cn deletion in SMCs, but not its phosphatase activity inhibition, diminished the contractile capacity of aortic and resistance arteries. These results are consistent with previous findings showing that impaired vessel contractility leads to diminished Ang-II-induced hypertension [17,34,36,37,52] and strongly suggest that SMC Cn regulates artery contraction and hypertension onset and maintenance in a phosphatase-activity-independent manner.

Cn deletion prevented not only Ang-II-induced hypertension, but also the BP increase induced by NE or L-NAME. It should be noted that these three stimuli increase [Ca^2+^]_i_, which is critical for VSMC contraction, and that Cn is implicated in the regulation of calcium dynamics [53,54]. Thus, one interesting possibility is that Cn might regulate BP by modulating [Ca^2+^]_i_ and therefore SM contraction. In this regard, a gene with the highest HS in the Harmonizome analysis was *Gja1*, which encodes connexin 43 (Cx43), a protein that regulates the Ca^2+^-dependent contraction of VSMCs [55].

Our results indicate that SMC Cn plays a key structural role in orchestrating the early induction of a gene expression program that includes 271 genes. While many of these genes are related to vessel contractility and hypertension onset and maintenance, nearly 30% of the genes whose regulation by Ang-II requires Cn expression but not its enzymatic activity have not been previously linked to hypertension and are thus potential novel targets for therapeutic intervention in hypertension.

The identification of *Serpine1* as one of the genes with the highest HS, whose regulation by Ang-II is mediated via Smad2 activation and the structural role of SMC Cn, is noteworthy. *Serpine1* encodes plasminogen activator inhibitor type-1 (PAI-1), whose plasma levels correlate with clinical hypertension [56]. *Serpine1* deletion has been shown to reduce L-NAME-induced hypertension [57]. Our present findings extend this knowledge by demonstrating that PAI-1 inhibition virtually abolished the contractile capacity of VSMCs and rapidly reverses the hypertension induced by Ang-II.

Although the ability of TM5441 to lower BP has been previously reported [58], our results reveal for the first time that TM5441 sharply and promptly reduces Ang-II-established hypertension. Specifically, the administration of TM5441 24 h after Ang-II-induced hypertension significantly reduced BP within 24 h, and acute administration after 7 days of Ang-II treatment fully reversed hypertension (Fig 8L–8O). Taken together, these findings highlight PAI-1 as a Cn-regulated mediator of hypertension and support its role as a critical factor in SMC contractility. Moreover, the data strongly advocate for the evaluation of PAI-1 inhibitors, such as TM5441, as potential therapeutic agents for hypertension treatment.

## Methods

### Mouse strains

To delete Cn specifically in SMCs or ECs, we crossed Cnb1^flox/flox^ (Cn^f/f^) mice [15] with either *Cdh5-Cre*^*ERT2*^ transgenic mice [16] to obtain EC-Cn^f/f^ mice or with *Myh11-Cre*^*ERT2*^ transgenic mice [17] to obtain SM-Cn^f/f^ mice. All mouse strains were backcrossed with C57BL/6J mice for more than eight generations to obtain a homogeneous genetic background and were maintained in homozygosity for the *CnB1*-floxed locus and in hemizygosity for the Cre^ERT2^ transgene in the case of EC-Cn^f/f^ and SM-Cn^f/f^ mice. All mice were genotyped by PCR of genomic DNA obtained from tail samples (genotyping PCR). In some experiments, genomic DNA samples from Tx-treated mice were analyzed by agarose gel electrophoresis to detect the Cre-loxP recombination (recombination PCR). Primer sequences used for genotyping were: *CnB1-*floxed mice (5′-CCCTAGGCACTGTTCATGGT-3′, 5′-GCCACAGATTAGCCTCGTGT-3′, 5′-CACATTCACCCACAATTGC-3′), *Myh11-Cre*^*ERT2*^ mice (5′-TGACCCCATCTCTTCACTCC-3′, 5′-AACTCCACGACCACCTCATC-3′, 5′-AGTCCCTCACATCCTCAGGTT-3′), and *Cdh5-Cre*^*ERT2*^ mice (5′-GCCTGCATTACCGGTCGATGCAACGA-3′, 5′-GTGGCAGATGGCGCGGCAACACCATT-3′).

### Animal procedures

Animal procedures were approved by the CNIC Ethics Committee and by the Madrid regional authorities (ref. PROEX 80/16 and PROEX 182.1/20) and conformed to European regulations regarding animal care (Directive EU 2010/63EU and Recommendation 2007/526/EC). All mice were housed in the CNIC specific pathogen-free animal facility with controlled temperature, humidity, and light-dark cycles. Sacrifice of mice was done by CO2 inhalation.

*Cnb1*^f/f^-Cre^ERT2-neg^ together with *Cnb1*^wt/wt^;*Cdh5*-Cre^ERT2^ or *Cnb1*^wt/wt^;*Myh11*-Cre^ERT2^, were used as controls (Cn-Ctl) for *Cnb1*^f/f^;*Cdh5*-Cre^ERT2^ or for *Cnb1*^f/f^;*Myh11*-Cre^ERT2^, respectively. For inducible and conditional gene deletion, 10–12-week-old Cn-Ctl, SM-Cn^f/f^, and EC-Cn^f/f^ male mice were given daily 1 mg i.p. injections of Tx (Sigma-Aldrich) on five consecutive days, generating SM-Cn^−/−^ and EC-Cn^−/−^ mice. Cn-Ctl, SM-Cn^−/−,^ and EC-Cn^−/−^ male mice were used after 6 weeks of Tx administration.

Ang-II (Sigma-Aldrich), CsA (Novartis), NE (Sigma-Aldrich), and TM5441 (MedChemExpress) were administered at 1 μg/kg/min, 5 mg/kg/d, 10 mg/kg/d, and 20 mg/kg/d, respectively, using subcutaneous osmotic minipumps (Alzet Corp, model 2001 or 2004). *N*_*ω*_-nitro-l-arginine methylester hydrochloride (l-NAME; 0.5 g/l; Sigma-Aldrich, N5751) was administered in drinking water every 3 days. For minipump implantation, all mice were isoflurane-anesthetized (3%–5% isoflurane for induction, 1%–2% isoflurane for maintenance), and a small surgical pocket was made in the dorsal skin, where the minipump was implanted. Mice undergoing the same surgical procedure but without minipump implantation were used as controls for the Cn-Ctl, SM-Cn^−/−^, EC-Cn^−/−^, and CsA-treated groups.

For the AAA model, Cn-Ctl, SM-Cn^−/−^, and EC-Cn^−/−^ male mice were fed a HFD (EF M HF coconut oil, + 7.5 g/kg Cholesterol Experimental diet, 38% chow, 10 mm; S9167-E011, SSNIFF) for 12 consecutive weeks. In the first week of feeding, Tx was injected to induce deletion in the Cre^ERT2^-expressing mice. After 12 weeks of the HFD, this treatment was combined with the systemic infusion (1 µg/kg/min) of Ang-II (Sigma-Aldrich) with osmotic minipumps (Alzet Corp) [19].

For the SM-Cn^−/−^ transcriptome analysis, short-term Ang-II infusion and mouse selection were performed as follows: male mice were treated with Tx and, after 6 weeks, baseline BP measurements were performed. Osmotic minipumps with Ang-II (1 µg/kg/min) were implanted in Cn-Ctl and SM-Cn^−/−^ mice in parallel, and BP was measured after 2 and 24 h. Treated Cn-Ctl mice with systolic BP > 130 mmHg were considered valid for analysis and were euthanized; all the SM-Cn^−/−^ mice treated in parallel were selected for analysis, since none of them showed increased BP after Ang-II infusion. For the CsA transcriptome analysis, osmotic minipumps with CsA (CsA group) were implanted in WT C57BL/6J male mice. Minipumps were not implanted in control mice at this stage. After 5 days, baseline BP measurements were taken. Ang-II osmotic minipumps (1 µg/kg/min) were implanted in CsA and control mice in parallel, and BP was measured after 2 and 24 h. Ang-II-treated CsA and control mice with a systolic BP > 130 mmHg were considered valid for analysis and euthanized. If we could not confirm *CnB1* deletion in any SM-Cn^−/−^ mouse or inhibition of NFAT dephosphorylation in the thymus of any CsA-treated mouse, those mice were removed from the experiment. Experiments for validation by RT-qPCR and protein expression by western blotting were performed in a similar way.

### Blood pressure measurements

Arterial BP was measured by the mouse tail-cuff method using the automated BP-2000 Blood Pressure Analysis System (Visitech Systems, Apex, NC, USA). In brief, mice were trained for BP measurements every day for one week. After training, BP was measured one day before treatment to determine the baseline BP values in each mouse cohort. Measurements were repeated several times during experiments. BP measurements were recorded in mice located in a tail-cuff restrainer over a warmed surface (37 °C). Fifteen consecutive systolic and diastolic BP measurements were made, and the last 10 readings per mouse were recorded and averaged.

### *In vivo* ultrasound imaging

Ultrasound images were obtained from isoflurane-anesthetized mice (1.5%–2% isoflurane) by high-frequency ultrasound with a VEVO 2100 echography device (VisualSonics, Toronto, Canada) at 30-micrometer resolution. Echocardiography was used to measure diastolic maximal internal aortic diameters and interventricular septum thickness in the parasternal long-axis view in the two-dimensional guided M-mode. Measurements were taken before treatment initiation to determine the baseline diameters and were repeated several times during the experiment. At endpoint, aortas were classified as AAA when they had increased in diameter by 50% or more [59,60], as dilated when the increase was 20%–50%, and as normal when the increase was 20% or less, relative to the initial measurement in all cases.

### Lentivirus production and infection

LV expressing Cre recombinase, GFP protein, LxVP, and LxVPmut peptides have been described previously [7,18]. Pseudo-typed LV production and titration was as described previously [18,61]. Briefly, HEK-293T cells were transiently transfected using the calcium phosphate method, and culture supernatant was concentrated by ultracentrifugation (2 h at 26,000 rpm; Ultraclear Tubes; SW28 rotor and Optima L-100 XP Ultracentrifuge; Beckman). Viruses were suspended in cold sterile phosphate-buffered saline (PBS) and titrated by transduction of Jurkat cells for 48 h. Transduction efficiency (GFP-expressing cells) and cell death (propidium iodide staining) were quantified by flow cytometry (BD FACSCanto Flow Cytometer and FlowJo v10.8 software). The HEK-293T and Jurkat cell lines were purchased from ATCC. All cells were mycoplasma-negative.

### Lentiviral transduction of aortas *in vivo*

WT male mice were anesthetized by intraperitoneal injection of ketamine (120 mg/kg) and xylazine (16 mg/kg), and a small incision was made to expose the jugular vein [7,61]. Virus solution (200 μl, 10^9^ particles/ml in PBS) was inoculated directly into the jugular vein 6 weeks before Ang-II minipump implantation. Transduction efficiency was analyzed postmortem in aortic samples by GFP immunohistochemistry.

### Cell procedures

Primary mouse VSMCs were isolated and grown as described [62]. In brief, aortas from Cn^f/f^ male mice were dissected, and the adventitia was removed with forceps. Aortic tissue was cut into small rings and digested with 1 mg/ml collagenase type II (Worthington) and 0.5 mg/ml elastase (Worthington) in DMEM (Gibco) until single cells were observed under the microscope. The enzymatic reaction was stopped by the addition of DMEM supplemented with bovine serum. For primary mAECs, digested tissue was incubated with anti-ICAM-2 (BD Biosciences, 553326) conjugated to magnetic beads (Dynal Thermofisher, 11035). Isolated cells were cultured, and the remaining tissue was removed on the following days.

VSMCs were cultured in DMEM (Gibco) supplemented with 20% FBS (Gibco) and antibiotics. mAECs were cultured in DMEM F-12 (BioWhittaker BE, 12-719F) supplemented with 20% FBS, 100 mg/ml heparin (Sigma-Aldrich), 5 mg/ml EC growth factor (Gibco), and antibiotics on culture plates pre-covered with 0.5% gelatin and 100 mg/ml collagen type I (Sigma-Aldrich, C2124-50ML). EC purity was > 80% after 1 week of culture. All experiments were performed in passages 3–5, and all cells were mycoplasma-negative.

VSMCs were infected with specific LVs for 5 h at a multiplicity of infection = 10. The medium was then replaced with fresh supplemented DMEM. Seven days after the transduction, infection efficiency was evaluated and experiments were performed. In the case of Cre-recombinase-expressing LVs, infection was confirmed by immunoblot detection of Cn deletion. In the case of LxVP-expressing LVs, infection was confirmed by RT-qPCR detection of GFP.

VSMCs were transfected with siRNA using Lipofectamine Transfection Reagent (Invitrogen, 13778075) following the manufacturer’s protocol. Smad2-specific siRNAs (ON-TARGETplus Mouse Smad2 siRNA (SMARTPool) L-040707-00-0005, Dharmacom) targeted the following sequences: 5′-ACUCAGAAUUGCAAUACUA-3′, 5′-CGAAUGUGCACCAUAAGAA-3′, 5′-GCUCAAGCAUGUCGUAAAC-3′, and 5′-GAAAGGGUUGCCACAUGUU-3′. Non-targeting control siRNAs (ON-TARGETplus Non-targeting Pool, D-001810-10-05, Dharmacom) targeted the sequences: 5′-UGGUUUACAUGUCGACUAA-3′, 5′-UGGUUUACAUGUUGUGUGA-3′, 5′-UGGUUUACAUGUUUUCUGA-3′, and 5′-UGGUUUACAUGUUUUCCUA-3′. Silencing efficiency was confirmed via immunoblot analysis, where Smad2 protein levels were evaluated in lysates from VSMCs transfected with Smad2 siRNAs. The results demonstrated effective knockdown of Smad2 expression, validating the transfection protocol.

For collagen contraction assays, 150 µl aliquots of PureColTM EZ Gel Solution (Sigma-Aldrich, 5074) were polymerized for 1 h at 37 °C in p48 wells, and 8 × 10^4^ VSMCs were seeded on each gel. Cells were left to adhere to the gels for 5 h and then starved for 72 h. The gel–cell composites were then gently detached from the plates and incubated in DMEM supplemented with 3% FBS for 24—48 h. When indicated, cells were pretreated with 50 µM TM5441 (MedChemExpress) for 2 h. At the end of the experiment, gels were washed in PBS and fixed with 3% paraformaldehyde (PFA) for 15 min. Gel images were taken with a Nikon SMZ800 scoop camera (0.8× objective). Each condition was replicated in 3–4 wells, and each condition was replicated with different batches of VSMCs. Gel contraction was quantified with ImageJ software as follows: each gel area was quantified and normalized to the average of all the areas in a single experiment; the means of the normalized areas for each condition are presented.

### Myography

Rings (3 mm long) from control (Cn-Ctl or WT) and SM-Cn^−/−^ male mouse thoracic descending aortas and superior MRA were excised and placed on a wire myograph (model 610M, Danish Myo Technology, Aarhus, Denmark) for the measurement of isometric tension. The organ chamber was filled with Krebs solution (composition in mM: NaCl 118, KCl 4.75, NaHCO_3_ 25, MgSO_4_ 1.2, CaCl_2_ 2, KH_2_PO_4_ 1.2, and glucose 11) and infused with carbogen (5% CO_2_ and 95% O_2_, pH ∼7.4), at 37 °C. When indicated, CsA (200 ng/ml) was added to WT arteries at the time of removal from the animal and was maintained during ring excision and throughout the experiment in the chamber. Aortic and MRA rings were stretched to tensions of 5 and 3 mN, respectively, and equilibrated for 30 min. Tension characteristics were obtained with the myograph software (LabChart). Aortic and MRA rings were initially stimulated with 80 and 50 mM KCl, respectively, followed by determination of KCl concentration-response curves (1–100 mM). Vasoconstriction measurements are presented as the force (mN) exerted by the rings.

### Aortic histology

After sacrifice of mice by CO_2_ inhalation, aortas were perfused with saline, isolated, and fixed in 4% PFA overnight at 4 °C. Paraffin cross sections (5 μm) from fixed aortas were stained with Masson’s trichrome (Masson), Hematoxylin and Eosin (HE), or Elastic Verhoeff–Van Gieson (EVG) stain. Medial wall thickness was determined from HE staining by quantifying the area of the media in four non-consecutive sections containing complete aortic cross-sections using the NDP.view2 Image viewing software.

For immunohistochemistry, deparaffinized sections (5 µm) were rehydrated, boiled to retrieve antigens (10 mM citrate buffer, pH 6), and blocked for 1 h with 5% goat serum plus 2% BSA in PBS. Samples were incubated with anti-GFP primary antibody (Invitrogen, A11122, 1:200) overnight at 4° and then with a biotinylated secondary antibody for 1 h at room temperature. Signal was detected with the DAB (3,3′-Diaminobenzidine) reaction (Vector Laboratories) for a maximum 7-min reaction time. Sections were then counterstained with hematoxylin, dehydrated, and mounted with DPX (CasaÁlvarez). For immunofluorescence, deparaffinized sections (5 µm) were rehydrated, boiled for antigen retrieval (10 mM citrate buffer, pH 6), and blocked for 1 h with 5% goat serum plus 2% BSA plus 5% horse serum in PBS-Triton 0.01% for anti-Ki67, anti-CD68, and anti- α-SMA. For p-Smad2, DNA was denatured with 1.5M HCl for 20 min RT, followed by blocking with 10% goat serum plus 2% BSA plus 10% horse serum in PBS-Triton 0.01%. Samples were incubated overnight at 4 °C with with the following primary antibodies: anti-Ki67 (ab15580, Abcam), anti-CD68 (ab15580, Abcam), and anti-phospho-Smad2 (Ser465/467; AB3849-I, Millipore) primary antibodies. Bound primary antibodies were detected with Alexa Fluor 594-conjugated or 647-conjugated secondary antibodies (Thermo Fisher, 1:500). Cy3 conjugated anti-α-SMA (C6198, Sigma, 1:2000) was incubated for 1 h RT. DAPI (1 ng/ml; Invitrogen) was used to label cell nuclei. Images were captured using a confocal microscope (Leica, SP5). For quantification, the medial layer of the aorta was defined as the region of interest (ROI) based on the elastin signal. ImageJ software was used to measure the mean intensity of α-SMA staining, and an ImageJ macro quantified nuclear p-Smad2 intensity. CD68-positive cells and Ki67-positive nuclei were manually counted and averaged per section. Data represent the average of at least two sections per mouse.

*In situ* southwestern histochemistry was performed as previously described [7]. Briefly, 5 μm aortic cross sections were deparaffinized and rehydrated. Sections were fixed with 0.5% PFA for 25 min at 28 °C, and endogenous alkaline phosphatase was quenched with 5 mM levamisole (L9756, Sigma-Aldrich). Preparations were then digested with 0.5% pepsin A (P7000, Sigma-Aldrich) in 1 N HCl for 35 min at 37 °C and washed twice with HEPES/BSA buffer (10 mM HEPES, 40 mM NaCl, 10 mM MgCl_2_, 1 mM dithiothreitol, 1 mM EDTA, and 0.25% BSA, pH 7.4). Sections were incubated with 0.1 mg/ml DNase I in HEPES/BSA buffer for 30 min at 30 °C. Activated NFAT proteins were probed with the synthetic sense DNA sequence 5′-GATCGCCCAAAGAGGAAAATTTGTTTCATACAG-3′. This sequence contains a composite site comprising the NFAT and AP1 sites from the mouse *IL-2* promoter [63]. The NFAT probe was labeled with digoxigenin (DIG) (Sigma-Aldrich) and diluted to 25 pM in HEPES/BSA buffer containing 0.5 mg/ml poly (dI-dC) (P4929, Sigma-Aldrich). Absence of probe was used as negative control. After overnight incubation at 37 °C in a humidified chamber, sections were washed twice with buffer 1 (0.1 M maleic acid, 0.15 M NaCl, 0.03% Tween-20, pH 7.5) and incubated for 1 h in blocking solution (0.1% SSC and 0.1% SDS diluted 1:10 in buffer 1 without Tween). The preparations were washed with buffer 1 and incubated for 2 h at 37 °C with an anti-DIG antibody conjugated to alkaline phosphatase (1:200 in blocking solution; 11093274910, Roche). Next, sections were washed in buffer 1 and buffer 3 (Tris HCl 0.1 M, 0.1 M NaCl, and 50 mM MgCl_2_, pH 9.5) at room temperature. Bound alkaline phosphatase was visualized with nitroblue tetrazolium chloride and 5-bromo-4-chloro-3-indolyl-phosphate (NBT/BCIP; 11681451001, Roche). The reaction was stopped by incubation in buffer 4 (10 mM Tris and 1 mM EDTA, pH 8.0), and sections were dehydrated through a graded ethanol series and mounted in 50% glycerol in PBS.

All bright-field images were obtained with a Leica DM2500 microscope with 20× and 40× HCX PL Fluotar objectives and with Leica Application Suite V3.5.0 software. Southwestern histochemistry images were analyzed and quantified with ImageJ software as follows: after color deconvolution by fast red blue DAB, the blue channel was selected (color 2). The same threshold was selected in all images, and only the medial aortic region was selected as a ROI and measured. Raw data are presented. Layouts were obtained in Adobe Illustrator.

### Immunoblotting

Mouse samples were obtained by dissection, and in the case of aortas, perivascular tissue and adventitia were removed with forceps. The tissue was frozen in liquid nitrogen and then homogenized using a mortar. Cultured cells were washed with ice-cold PBS and frozen at −80 °C. Protein extracts from tissue samples and cells were obtained in ice-cold high-salt lysis buffer (20 mM Tris HCl pH 7.5, 5 mM MgCl_2_, 50 mM NaF, 10 mM EDTA, 0.5 M NaCl, 1% Triton X-100) for the detection of CnB, CnA, tubulin, α-SMA, Cd31, and NFATc3, and in RIPA buffer (150 mM NaCl, 10mM Tris HCl pH8, 1% Triton X-100, 0.1% SDS, 1% sodium deoxycholate and 5 mM EDTA) for the detection of PAI-1, phospho-Smad2 (Ser465/467), Smad2/3 and tubulin. Both lysis buffers were supplemented with protease, phosphatase, and kinase inhibitors. Homogenates were centrifuged for 15 min at 16,000*g* at 4 °C, and proteins were collected in the supernatants. Protein was quantified with the Lowry assay kit. Protein samples (30 μg) were separated under reducing conditions on SDS-polyacrylamide gels and transferred to nitrocellulose membranes. Proteins were detected with the following primary antibodies: mouse monoclonal anti-CnB (Sigma, 0581, 1:1000), rabbit polyclonal anti-CnA (Merck Millipore, 07-1491, 1:2000), mouse monoclonal anti-tubulin (Sigma, T6074, 1:40000), rabbit polyclonal anti-Cd31 (Abcam, ab28364, 1:1000), mouse monoclonal anti α-SMA (Sigma, A5228; 1:5000), rabbit polyclonal anti-NFATc3 (M-75) (SCB, sc-8321, 1:1000), rabbit monoclonal anti-PAI-1 (Abcam, ab222754, 1:500), anti-phospho-Smad2 (Cell Signaling 3108, 1:1000), and anti-Smad2/3 (Cell Signaling, 8685, 1:1000). After washing and incubation with the appropriate specific HRP-conjugated secondary antibodies, immunoreactive bands were visualized using the enhanced chemiluminescence (ECL) detection reagent (GE Healthcare, Chicago, IL, USA, RPN2106) or acquired in the ImageQuant LAS 4000 device using the Immobilon Forte Western HRP substrate (WBLUF0100, Millipore). Quantification of protein band intensity was performed with ImageJ using Tubulin band intensity for normalization.

### Reverse transcription and quantitative PCR

Aortas were extracted after perfusion with 5 ml saline solution, and the adventitia layer was discarded. Frozen tissue was homogenized using a mortar and an automatic bead homogenizer (MagNA Lyzer). Total RNA was isolated with TRIZOL (Life Technologies). Total RNA (1 μg) was reverse transcribed at 37 °C for 50 min in a 20 µl reaction mix containing 200U Moloney murine leukemia virus (MMLV) reverse transcriptase (Life Technologies), 100 ng random primers, and 40U RNase Inhibitor (Life Technologies). The resulting cDNA was analyzed by qPCR in an AB7900 sequence detection PCR system (Applied Biosystem, Foster City, CA, USA) using SYBR Green PCR or TaqMan Universal PCR master mixes (Go Taq Probe PCR Master Mix; Promega, A600A or A610A, respectively). The following TaqMan probes were used: *Ptgs2* (Mm00478374_m1) and *Hprt1* (Mm00446968_m1). The specific probes used for SYBR Green detection were as follows: *Gja1* (5′-CTTGGGGTGATGAACAGT-3′; 5′-TGAGCCAAGTACAGGAGT-3′ [64]), *Hprt* (5′-GCTGGTGAAAAGGACCTCT-3′; 5′-CACAGGACTAGAACACCTGC-3′), *Ier3* (5′-GCGCGTTTGAACACTTCTCG-3′; 5′-TGGCGCCGGACCACTC-3′), *Rcan1–4* (5′-TTGTGTGGCAAACGATGATGT-3′; 5′-CCCAGGAACTCGGTCTTGT-3′), *Serpine1* (5′-GAGGTAAACGAGAGCGGCA-3′; 5′-AAGAGGATTGTCTCTGTCGGG-3′). Probe specificity was checked by post-amplification melting-curve analysis in the case of SYBR amplification; for each reaction, only one Tm peak was produced. The amount of target mRNA in samples was estimated by the 2^−∆CT^ relative quantification method, using *Hprt* for normalization, and represented in graphs. Each sample represented belongs to one mouse aorta.

### RNA sequencing

Aortas were isolated after perfusion with saline, and the perivascular tissue and the adventitia layer were removed with forceps. A piece of aortic tissue was used for PCR confirmation of *CnB1* recombination, and the thymus was isolated for protein extraction to check the NFAT phosphorylation state, when appropriate. Aortic tissue was frozen in liquid nitrogen and homogenized with lysis buffer (Qiagen, 64204) using an automatic bead homogenizer (MagNA Lyser; Roche). Total RNA was isolated with the RNeasy MinElute Cleanup Kit (QIAGEN, 74204). Each triplicate was a pool of three aortas (total, nine aortas per condition). RNA quality and purity were analyzed by Nanodrop and Bioanalyzer (Agilent 2100 Bioanalyzer RNA NanoChip). Only RNA samples with appropriate absorbance ratios were used (260/280 nm > 2.0; 230/260 nm 2.0–2.2); RNA samples with a RIN (RNA Integrity number-Bioanalyzer) below seven were discarded. For each condition, 1.5 μg of RNA was retro-transcribed with oligo dT priming, and the cDNA obtained was used to generate the transcriptome library. The library was sequenced using Illumina technology (Illumina Genome Analyzer IIx System). Library generation and sequencing were performed by the CNIC Genomics Unit under the supervision of Dr. Ana Dopazo. Reads were aligned first using BoWTie version 0.12.7 and then mapped to the latest Ensembl transcript set using RSEM v1.16. One replicate of the untreated Cn-Ctl group showed a high deviation from the other two replicates in PCA analysis and was therefore discarded. Genes showing altered expression with an adjusted *p* < 0.05 were considered DEGs. Only protein-coding genes were included in further analysis. Gene expression data are available at http://www.ncbi.nlm.nih.gov/projects/geo/ under accession number GSE255061.

Venn diagrams were generated with the online software Venny 2.1.0 (https://bioinfogp.cnb.csic.es/tools/venny/index.html), and the DEGs represented in each diagram are indicated in the figure and figure legend. Heatmaps were generated using R library ‘stats’ [65] and ‘gplots’ [66] to represent mean normalized mRNA expression of the DEGs regulated by Ang-II in Cn-Ctl, SM-Cn^−/−^, WT, and CsA-pretreated mice versus the untreated condition (FC > ±2). Mean normalized mRNA expression was obtained from triplicates of each condition. The HS was extracted automatically by an exact pattern match using the gen symbol as the identifier. The processed database was the Harmonizome dataset collection [67] for the curated CTD Gene-Disease Associations related to the term ‘Hypertension’, where the standardized value was renamed the HS.

### Functional enrichment analysis

Gene Ontology (GO) enrichment analysis of DEGs was performed using g:Profiler software [68]. The analysis mainly focused on gen function data sources related to cellular components (GO:CC). We were able to map genes to statistically significant enriched terms. The statistical domain scope was only applied to annotated genes, adjusting the significance threshold by multiple testing (using the g:Profiler tailor-made algorithm g:SCS), set to a value *p* < 0.05. This approach has produced more accurate results than obtained with the standard Bonferroni correction or BH-False Discovery Rate methodologies [69]. To select the most relevant functional categories, the term size was adjusted to 0 as minimum and 300 as maximum, avoiding redundant and generic terms.

### Statistical analysis

GraphPad Prism software (versions 9.4.1 and 10.1.2) was used for the statistical analysis. Appropriate tests were chosen according to the data distribution based on the Saphiro–Wilk normality test. Outliers were identified and excluded by the ROUT method.

Comparisons for measured parameters and repeated measurements (RM) are indicated in each figure legend. Differences were analyzed by unpaired Student *t* test, Mann-Whitney test, and one-way ANOVA or two-way ANOVA tests, and multiple comparisons were analyzed with the Tukey or Šídák post hoc tests, as appropriate; the tests used are indicated in each figure legend. Differences were considered statistically significant at *p* < 0.05.

The number of animals used is indicated in the corresponding figures or figure legends. No statistical method was used to predetermine sample size. Sample size was chosen empirically according to our experience in the calculation of experimental variability. All experiments were carried out with at least three biological replicates.

Experimental groups were balanced in terms of animal age and weight. Only male mice were used because the *Myh11-Cre*^*ERT2*^ transgene was inserted in the Y chromosome (https://www.jax.org/strain/019079). Investigators were not blinded to group allocation during experiments or to outcome assessments. Animals were genotyped before experiments, caged together, and treated in the same way.

## Supporting information

S1 Fig Ang-II administration in mice does not modify Cn expression in the aorta. (**A**) Representative immunoblot analysis of CnA, CnB and tubulin (loading control) and (**B**) quantification of their relative expression in protein extracts from untreated Cn-Ctl (*n* = 3–4) and Ang-II-treated Cn-Ctl (*n* = 4–7), Molecular weights (kDa) are indicated. Each data point denotes an individual mouse, and data in histograms are presented as mean ± s.e.m. unpaired Student *t* test. Underlying data can be found in S1 Data.
(TIF)

S2 Fig Cn deletion in SMCs does not prevent HFD-induced body weight gain. Body weight of 29 Cn-Ctl, 19 EC-Cn^−/−^, and 28 SM-Cn^−/−^ mice fed a chow diet and 46 Cn-Ctl, 15 EC-Cn^−/−^, and 18 SM-Cn^−/−^ mice after 12 weeks of HFD. Each data point denotes an individual mouse, and the horizontal bars denote the mean (long bar) and the s.e.m. ****p < 0.0001, ***p < 0.001; one-way ANOVA with Tukey’s multiple comparison post hoc test. Underlying data can be found in S1 Data.
(TIF)

S3 Fig Immunofluorescence characterization of the AbAo in the AAA mouse model. (**A**) Representative images of α-SMA immunostaining in AbAo cross-sections from the indicated groups of mice, and (**B**) quantification of α-SMA mean intensity in the same cross-sections. Representative images of (**C**) Ki67 (arrowheads), and (**D**) CD68 (arrowheads) immunostaining in AbAo cross-sections from the indicated groups of mice, with quantification of (**E**) Ki67 + nuclei and (**F**) CD68+  cells in the same cross-sections. (A,C,D) L, lumen. Scale bar, 100 μm. (B,E,F) Each data point denotes an individual mouse, with values representing the average quantification of at least 2 independent images per mouse. Data in histograms are presented as mean ± s.e.m. ***p < 0.001, **p < 0.01, *p < 0.05; two-way ANOVA with Šídák’s post hoc test (B); Mann–Whitney and unpaired Student *t* test (E, F). Underlying data can be found in S1 Data.
(TIF)

S4 Fig Cn deletion in SMCs inhibits AngII-induced diastolic hypertension. Diastolic BP values at the indicated times in 5 Cn-Ctl, 3 EC-Cn^−/−^, and 5 SM-Cn^−/−^ control-treated mice and 22 Cn-Ctl, 10 EC-Cn^−/−^, and 18 SM-Cn^−/−^ Ang-II-treated mice. Data are mean ± s.e.m. ****p < 0.0001, ***p < 0.001, **p < 0.01 vs. SM-Cn^−/−^ Ang-II, ^###^*p* < 0.001 vs. Cn-Ctl control, ^##^*p* < 0.01, ^#^*p* < 0.05 vs SM-Cn^−/−^ or Cn-Ctl control; RM two-way ANOVA with Tukey’s post hoc test. Underlying data can be found in S1 Data. Data from untreated and Ang-II-treated Cn-Ctl mice were repeated in left and right panels as indicated in S1 Data.
(TIF)

S5 Fig Cn deletion in SMCs inhibits norepinephrine (NE)- and L-NAME-induced AHT. (**A**) Experimental design: 10–12-week-old mice were treated with tamoxifen for 5 consecutive days (open arrow heads) and, after 6 weeks, NE osmotic minipumps were implanted for 4 weeks in one group of mice; control mice were operated without minipump implantation. Another mouse group was treated with L-NAME in drinking water for 4 weeks. BP was measured at the indicated time points (purple arrows), and mice were euthanized at the end of the experiment. (**B**) Systolic BP values of 10 Cn-Ctl and 5 SM-Cn^−/−^ control-treated mice, 11 Cn-Ctl and 6 SM-Cn^−/−^ NE-treated mice, and 5 Cn-Ctl and 6 SM-Cn^−/−^ L-NAME-treated mice. Data are means ± s.e.m. ***p < 0.001, **p < 0.01, *p < 0.05 vs. treated SM-Cn^−/−^, ^####^*p* < 0.001 vs. control Cn-Ctl and ^#^*p* < 0.05 vs. control SM-Cn^−/−^; RM two-way ANOVA with Tukey’s post hoc test. Underlying data can be found in S1 Data. Data from untreated Cn-Ctl and untreated SM-Cn^−/−^ mice are repeated in top and bottom panels as indicated in S1 Data.
(TIF)

S6 Fig Cn inhibition by CsA impairs Ang-II-induced NFAT dephosphorylation and cardiac hypertrophy. (**A**) Representative NFATc3 immunoblot analysis of thymus extracts from WT mice treated as indicated (top panel) and Ponceau staining of the membrane (bottom panel). Hyper-phosphorylated NFATc3 (P-NFAT) and de-phosphorylated NFATc3 (NFAT) are indicated. (**B**) End-of-experiment heart weight vs. body weight ratio (HW/BW) values. Each data point denotes an individual mouse, and data in histograms are presented as mean ± s.e.m. ****p < 0.0001, ***p < 0.001; two-way ANOVA with Šídák’s post hoc test. (**C**) Echocardiography-determined interventricular septum (IVS) thickness before CsA (day −2) and Ang-II (day 0) administration and after 21 days of Ang-II treatment. Each data point denotes an individual mouse, and the horizontal bars denote the mean (long bar) and the s.e.m. ****p < 0.0001, **p < 0.01; RM two-way ANOVA with Tukey’s post hoc test. (B, C) Ang-II (*n* = 10), CsA + Ang-II (*n* = 8), control (*n* = 8), and CsA (*n* = 5). Underlying data can be found in S1 Data.
(TIF)

S7 Fig Cn deletion, but not inhibition of Cn phosphatase activity, prevents Ang-II-induced diastolic hypertension. (**A, B**) Diastolic BP was measured at the indicated time points in the same Cn-Ctl, SM-Cn^−/−^, and non-pretreated and CsA-pretreated WT mice as in Fig 6B (A) and Fig 6D (B). Each data point denotes the BP value in an individual mouse, and the horizontal bars denote the mean (long bar) and the s.e.m. (*n* = 9 mice per group and per time point). ****p < 0.0001, **p < 0.01, *p < 0.05 vs. baseline; RM two-way ANOVA with Tukey’s post hoc test. ^##^*p* < 0.01 vs. 2 or 24 h Ang-II Cn-Ctl; RM two-way ANOVA with Šídák’s post hoc test. (**C**) Representative NFATc3 immunoblot of thymus extracts from mice treated as indicated. Hyper-phosphorylated NFATc3 (P-NFAT) and de-phosphorylated NFATc3 (NFAT) are indicated. Ponceau staining of the membrane is shown below. (**D**) Quantification of mRNA expression, as assessed by RT-qPCR, in extracts from the aorta of untreated control (3 Cn-Ctl plus 3 WT), SM-Cn^−/−^ (*n* = 5), and CsA-pretreated (*n* = 6) mice and Ang-II-treated control (3 Cn-Ctl plus 3 WT), SM-Cn^−/−^ (*n* = 5), and CsA-pretreated (*n* = 6) mice. Each data point denotes an individual mouse, and data in histograms are presented as mean ± s.e.m. **p < 0.01, ****p < 0.0001; two-way ANOVA with Šídák’s post hoc test. Underlying data can be found in S1 Data.
(TIF)

S8 Fig Selection of potentially AHT-related genes from the transcriptomic analyses. (**A**) 800 DEGs in Cn-Ctl mice treated with Ang-II for 2 h were selected as potential mediators of AHT, and 130 DEGs regulated in SM-Cn^−/−^ mice treated with Ang-II for 2 h were excluded, leaving 722 potentially AHT-related genes, as indicated in the Venn diagram (below). (**B**) Of these genes, only those whose expression differed between the hypertensive condition (Cn-Ctl Ang-II 2 h) and the non-hypertensive condition (SM-Cn^−/−^ Ang-II 2 h) were selected using a Venn diagram (below). The figure indicates the resulting 336 DEGs potentially involved in AHT and whose regulation is Cn-expression dependent. (**C**) 2,653 DEGs regulated in WT mice by treatment with Ang-II for 2 h were selected as potential mediators of AHT. Only those whose expression was similar in both hypertensive conditions (not showing differential expression between WT Ang-II 2 h and CsA Ang-II 2 h) and CsA-independent were selected using a Venn diagram (below). The figure indicates the resulting 2,596 DEGs potentially involved in AHT and whose regulation is independent of Cn phosphatase activity.
(TIF)

S9 Fig Collagen contraction assay. (**A**) Representative images from three independent experiments and (**B**) quantification of the surface area of fixed collagen gels in the absence or presence of cells stimulated as indicated. Each data point denotes the mean of each experiment, and data in histograms are presented as mean ± s.e.m. *p < 0.05 by two-way ANOVA with Šídák’s post hoc test. No cells without serum (*n* = 2), starved cells (*n* = 3), No cells with 3% FBS (*n* = 3), Cells with 3% FBS (*n* = 3). Underlying data can be found in S1 Data.
(TIF)

S10 Fig *Serpine1*, *Gja1*, and *Ier3* induction is mediated by a structural role of SMC Cn. RT-qPCR analysis of mRNA expression in aortic extracts from untreated control (3 Cn-Ctl plus 3 WT), SM-Cn^−/−^ (*n* = 5), and CsA-pretreated (*n* = 6) mice and from Ang-II-treated control (3 Cn-Ctl plus 3 WT), SM-Cn^−/−^ (*n* = 5), and CsA-pretreated (*n* = 6) mice. Two-way ANOVA with Šídák post hoc test; ****p < 0.0001, ***p < 0.001, **p < 0.01, *p < 0.05. Underlying data can be found in S1 Data.
(TIF)

S11 Fig Smad2 activation is mediated by a structural role of SMC Cn. (**A**) Representative immunoblot analysis of phospho-Smad2, Smad2/3 and tubulin (loading control) and (**B**) quantification of their relative expression in protein extracts from untreated control (*n* = 3) and SM-Cn^−/−^ (*n* = 3) mice; CsA-pretreated (*n* = 3) mice; and Ang-II-treated control (*n* = 4), SM-Cn^−/−^ (*n* = 4), and CsA-pretreated (*n* = 4) mice. Molecular weights (kDa) are indicated. Each data point denotes an individual mouse, and data in histograms are presented as mean ± s.e.m. *p < 0.05, **p < 0.01, ***p < 0.001, ****p < 0.0001; two-way ANOVA with Šídák post hoc test. (**C**) Quantification of the relative expression in protein extracts from the representative immunoblot in Fig 8F (*n* = 3 independent experiments) with each data point representing an individual replicate. Data are presented as mean ± s.e.m. ***p < 0.001, unpaired Student *t* test. Underlying data can be found in S1 Data.
(TIF)

S1 Table List of the 722 DEGs indicated in S8A Fig. (XLSX)

S2 Table List of the 336 DEGs indicated in S8B Fig. (XLSX)

S3 Table List of the 2,653 DEGs indicated in S8C Fig. (XLSX)

S4 Table List of the 271 DEGs indicated in Fig 7C. (XLSX)

S1 Raw Images Original, uncropped images supporting all blot results in article’s figures and Supporting information files. (PDF)

S1 Data Data underlying Figs 1–8 and S1–S11. (XLSX)

## Abbreviations

**:**
AA: aortic aneurysm
AAA: abdominal aortic aneurysm
AbAo: abdominal aorta
Ang-II: angiotensin-II
AsAo: ascending aorta
BP: blood pressure
Cn: calcineurin
CsA: cyclosporine A
DEGs: differentially expressed genes
DIG: digoxigenin
EC: endothelial cell
ECM: extracellular matrix
EVG: Elastic Verhoeff–Van Gieson
FC: fold change
GO: Gene Ontology
HE: Hematoxylin and Eosin
HFD: high-fat diet
HS: hypertension score
LV: lentivirus
mAECs: mouse aortic endothelial cells
MRA: mesenteric resistance artery
NE: norepinephrine
PAI-1: plasminogen activator inhibitor type-1
PBS: phosphate-buffered saline
PFA: paraformaldehyde
RT-qPCR: reverse transcription quantitative real-time polymerase chain reaction
RM: repeated measurements
ROI: region of interest
SMC: smooth muscle cell
Tx: tamoxifen
VSMCs: vascular smooth muscle cells
VWR: vascular wall remodelling
