# Supplementary figures and images for: Phosphatase-independent activity of smooth-muscle calcineurin orchestrates a gene expression program leading to hypertension
Source: PLoS Biol. 2025 May 14;23(5):e3003163. doi: 10.1371/journal.pbio.3003163 (PMC12165594; doi:10.1371/journal.pbio.3003163)

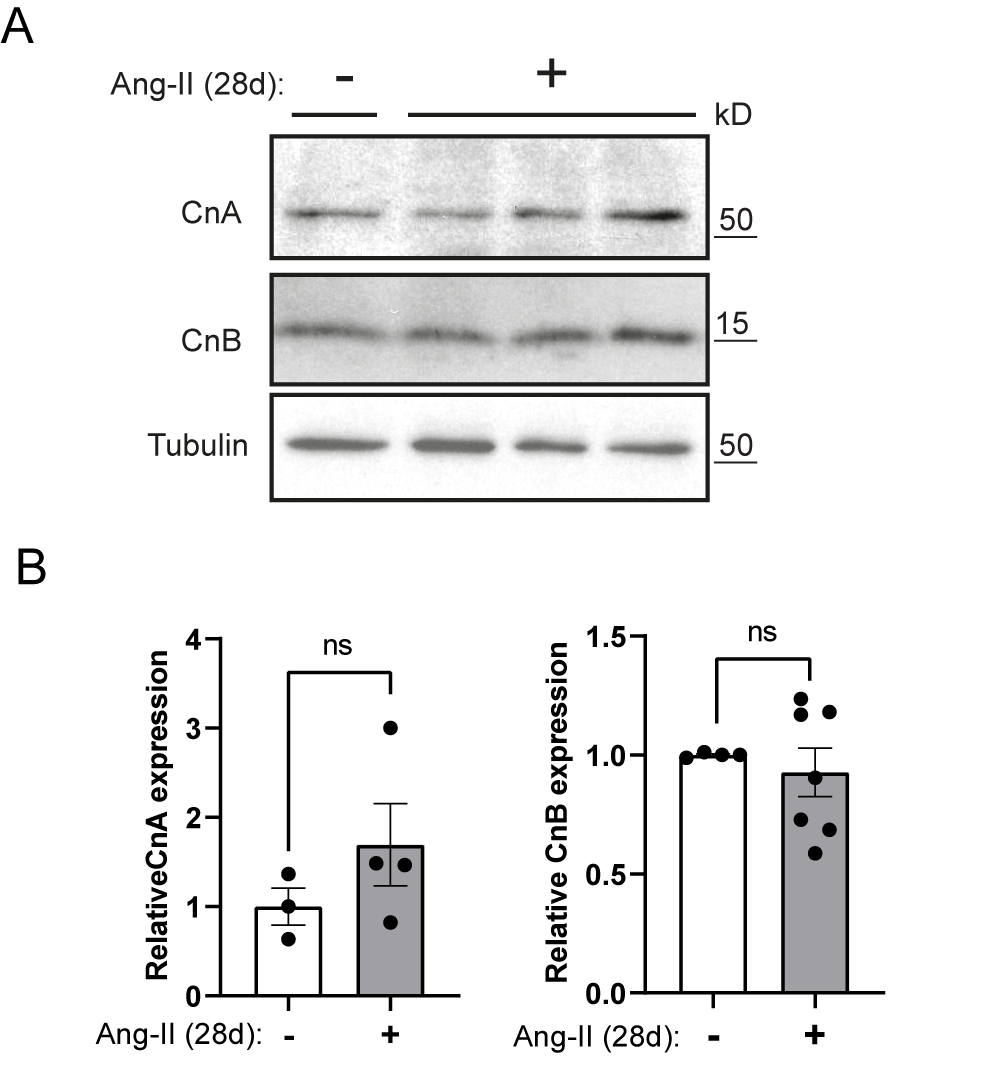

Supplement: S1 Fig — (A) Representative immunoblot analysis of CnA, CnB and tubulin (loading control) and (B) quantification of their relative expression in protein extracts from untreated Cn-Ctl (n = 3–4) and Ang-II-treated Cn-Ctl (n = 4–7), Molecular weights (kDa) are indicated. Each data point denotes an individual mouse, and data in histograms are presented as mean ± s.e.m. unpaired Student t test. Underlying data can be found in S1 Data. (TIF) [file pbio.3003163.s001.tif]

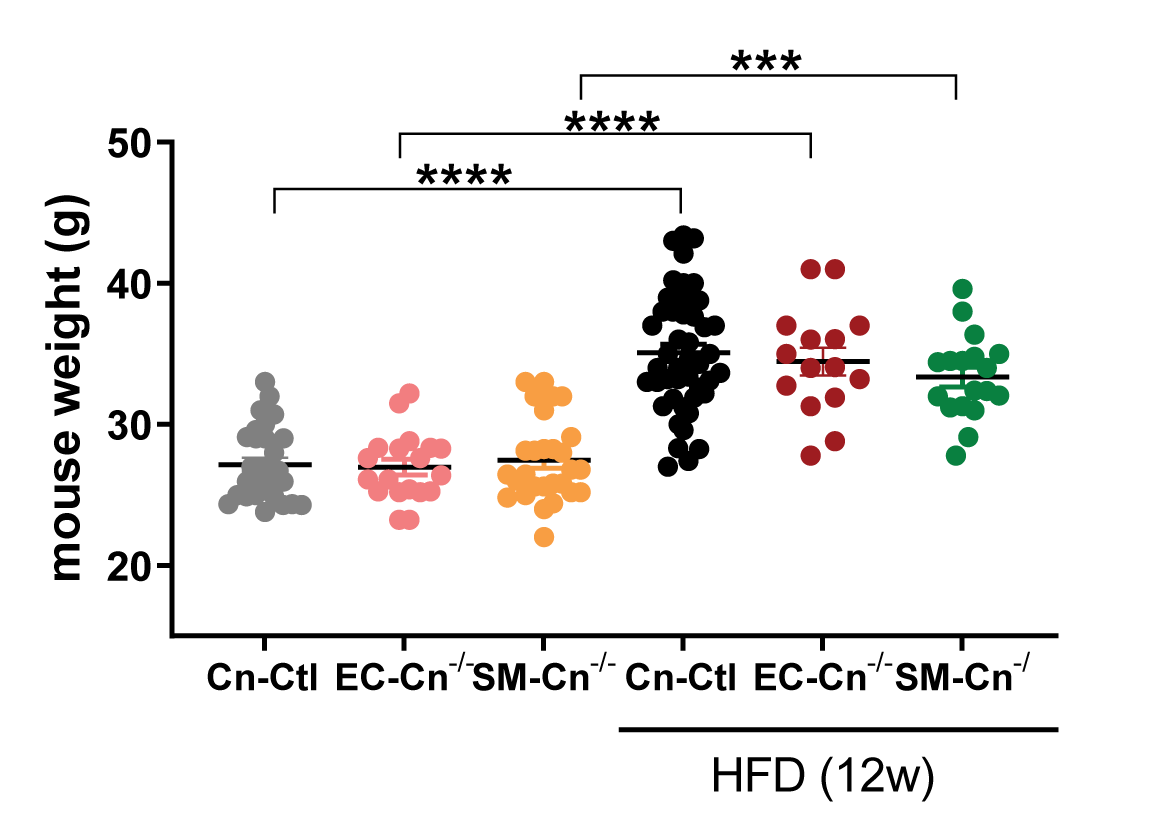

Supplement: S2 Fig — Body weight of 29 Cn-Ctl, 19 EC-Cn−/−, and 28 SM-Cn−/− mice fed a chow diet and 46 Cn-Ctl, 15 EC-Cn−/−, and 18 SM-Cn−/− mice after 12 weeks of HFD. Each data point denotes an individual mouse, and the horizontal bars denote the mean (long bar) and the s.e.m. ****p < 0.0001, ***p < 0.001; one-way ANOVA with Tukey’s multiple comparison post hoc test. Underlying data can be found in S1 Data. (TIF) [file pbio.3003163.s002.tif]

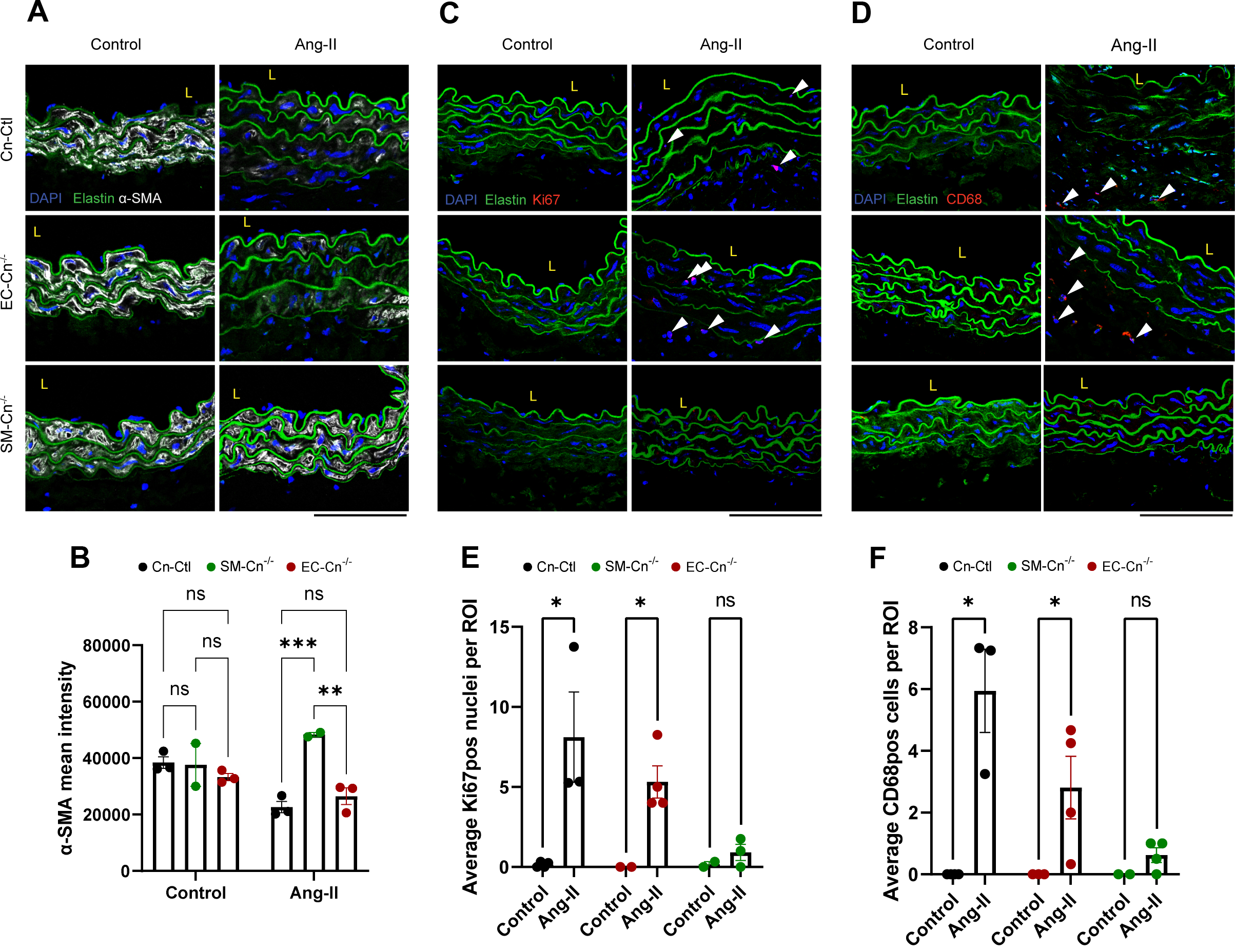

Supplement: S3 Fig — (A) Representative images of α-SMA immunostaining in AbAo cross-sections from the indicated groups of mice, and (B) quantification of α-SMA mean intensity in the same cross-sections. Representative images of (C) Ki67 (arrowheads), and (D) CD68 (arrowheads) immunostaining in AbAo cross-sections from the indicated groups of mice, with quantification of (E) Ki67 + nuclei and (F) CD68+ cells in the same cross-sections. (A,C,D) L, lumen. Scale bar, 100 μm. (B,E,F) Each data point denotes an individual mouse, with values representing the average quantification of at least 2 independent images per mouse. Data in histograms are presented as mean ± s.e.m. ***p < 0.001, **p < 0.01, *p < 0.05; two-way ANOVA with Šídák’s post hoc test (B); Mann–Whitney and unpaired Student t test (E, F). Underlying data can be found in S1 Data. (TIF) [file pbio.3003163.s003.tif]

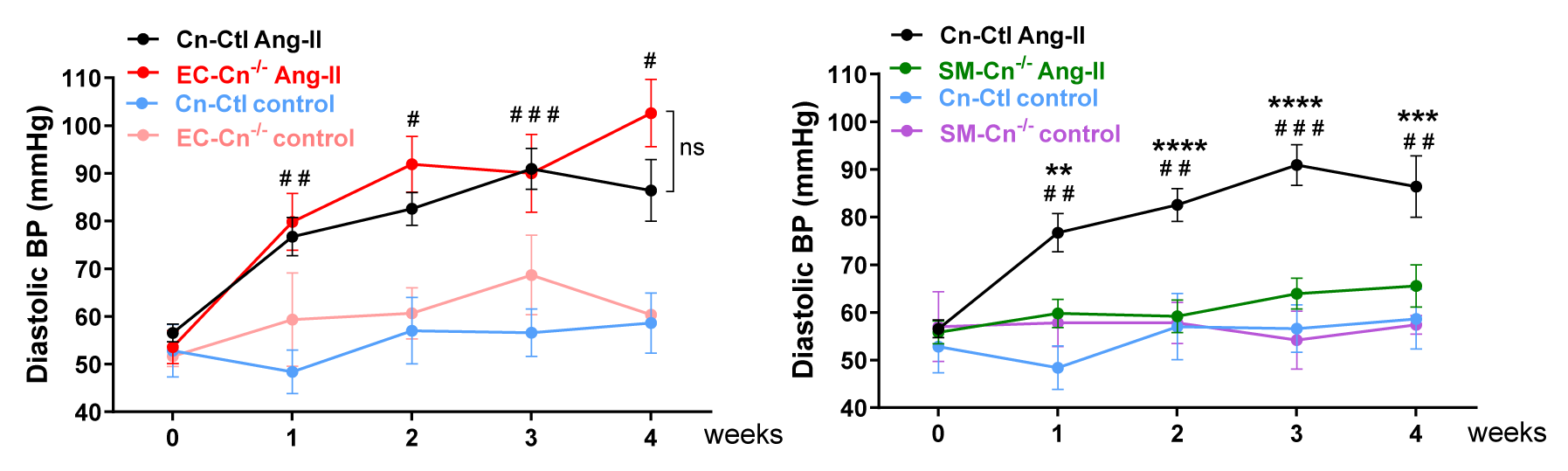

Supplement: S4 Fig — Diastolic BP values at the indicated times in 5 Cn-Ctl, 3 EC-Cn−/−, and 5 SM-Cn−/− control-treated mice and 22 Cn-Ctl, 10 EC-Cn−/−, and 18 SM-Cn−/− Ang-II-treated mice. Data are mean ± s.e.m. ****p < 0.0001, ***p < 0.001, **p < 0.01 vs. SM-Cn−/− Ang-II, ###p < 0.001 vs. Cn-Ctl control, ##p < 0.01, #p < 0.05 vs SM-Cn−/− or Cn-Ctl control; RM two-way ANOVA with Tukey’s post hoc test. Underlying data can be found in S1 Data. Data from untreated and Ang-II-treated Cn-Ctl mice were repeated in left and right panels as indicated in S1 Data. (TIF) [file pbio.3003163.s004.tif]

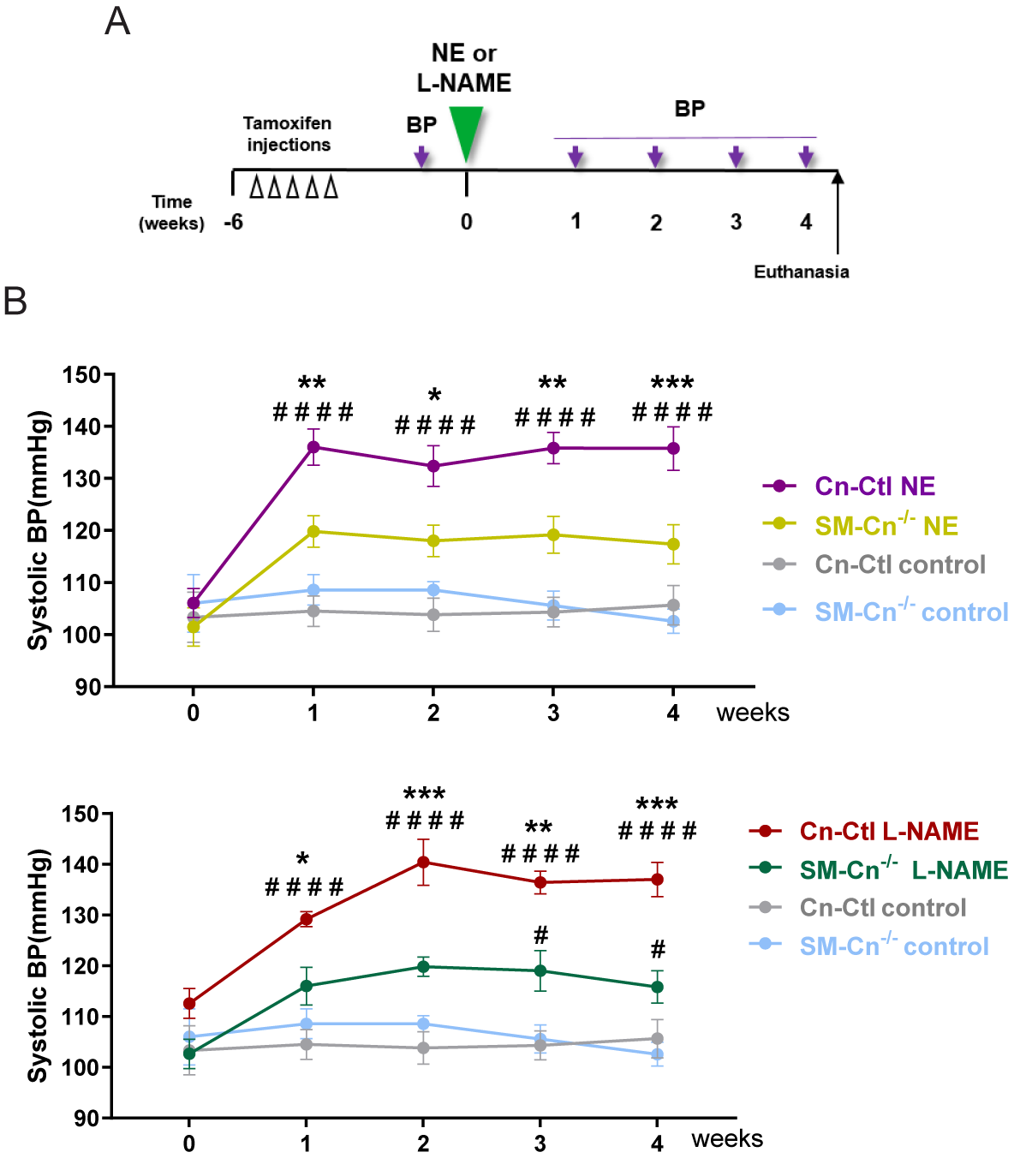

Supplement: S5 Fig — (A) Experimental design: 10–12-week-old mice were treated with tamoxifen for 5 consecutive days (open arrow heads) and, after 6 weeks, NE osmotic minipumps were implanted for 4 weeks in one group of mice; control mice were operated without minipump implantation. Another mouse group was treated with L-NAME in drinking water for 4 weeks. BP was measured at the indicated time points (purple arrows), and mice were euthanized at the end of the experiment. (B) Systolic BP values of 10 Cn-Ctl and 5 SM-Cn−/− control-treated mice, 11 Cn-Ctl and 6 SM-Cn−/− NE-treated mice, and 5 Cn-Ctl and 6 SM-Cn−/− L-NAME-treated mice. Data are means ± s.e.m. ***p < 0.001, **p < 0.01, *p < 0.05 vs. treated SM-Cn−/−, ####p < 0.001 vs. control Cn-Ctl and #p < 0.05 vs. control SM-Cn−/−; RM two-way ANOVA with Tukey’s post hoc test. Underlying data can be found in S1 Data. Data from untreated Cn-Ctl and untreated SM-Cn−/− mice are repeated in top and bottom panels as indicated in S1 Data. (TIF) [file pbio.3003163.s005.tif]

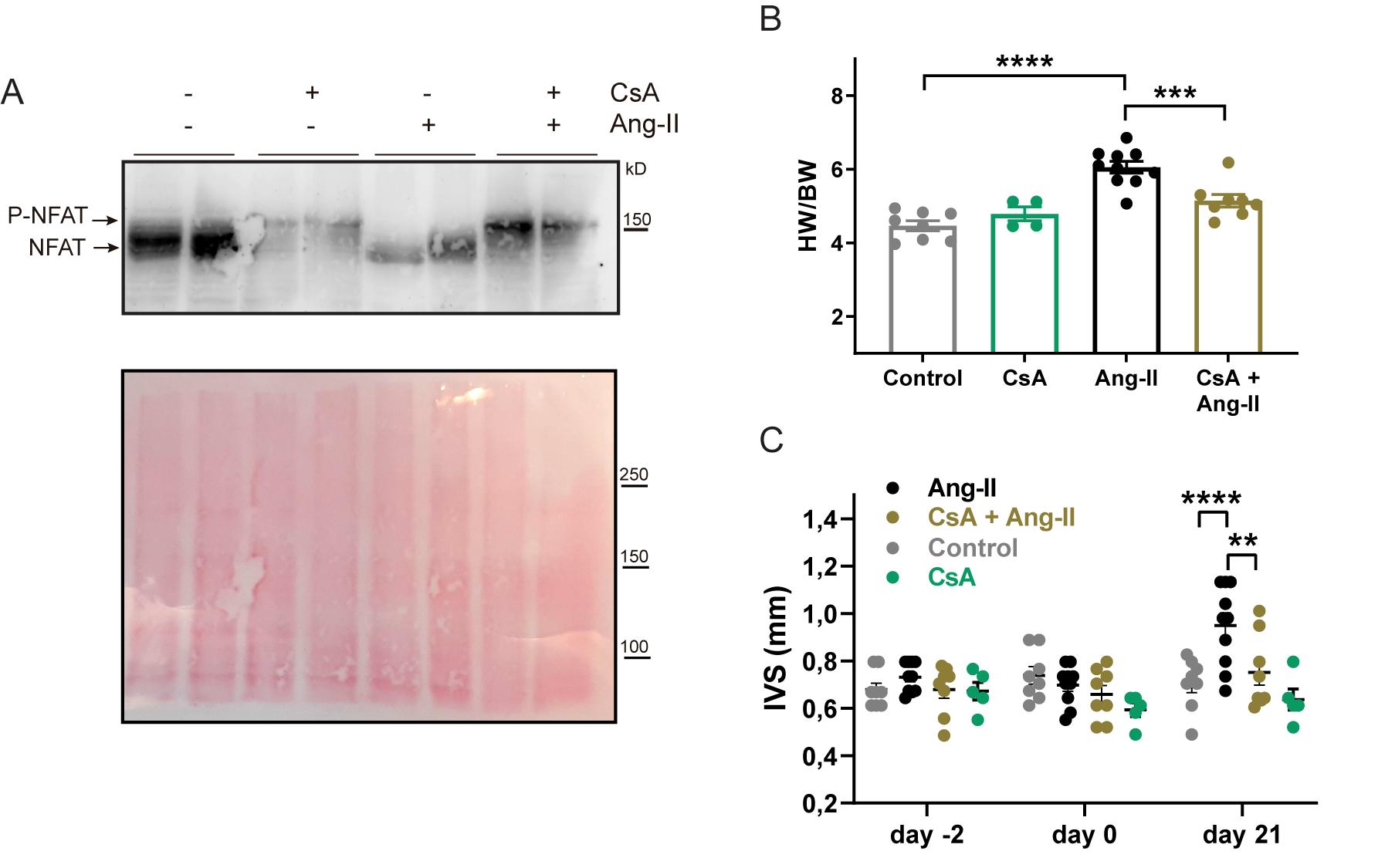

Supplement: S6 Fig — (A) Representative NFATc3 immunoblot analysis of thymus extracts from WT mice treated as indicated (top panel) and Ponceau staining of the membrane (bottom panel). Hyper-phosphorylated NFATc3 (P-NFAT) and de-phosphorylated NFATc3 (NFAT) are indicated. (B) End-of-experiment heart weight vs. body weight ratio (HW/BW) values. Each data point denotes an individual mouse, and data in histograms are presented as mean ± s.e.m. ****p < 0.0001, ***p < 0.001; two-way ANOVA with Šídák’s post hoc test. (C) Echocardiography-determined interventricular septum (IVS) thickness before CsA (day −2) and Ang-II (day 0) administration and after 21 days of Ang-II treatment. Each data point denotes an individual mouse, and the horizontal bars denote the mean (long bar) and the s.e.m. ****p < 0.0001, **p < 0.01; RM two-way ANOVA with Tukey’s post hoc test. (B, C) Ang-II (n = 10), CsA + Ang-II (n = 8), control (n = 8), and CsA (n = 5). Underlying data can be found in S1 Data. (TIF) [file pbio.3003163.s006.tif]

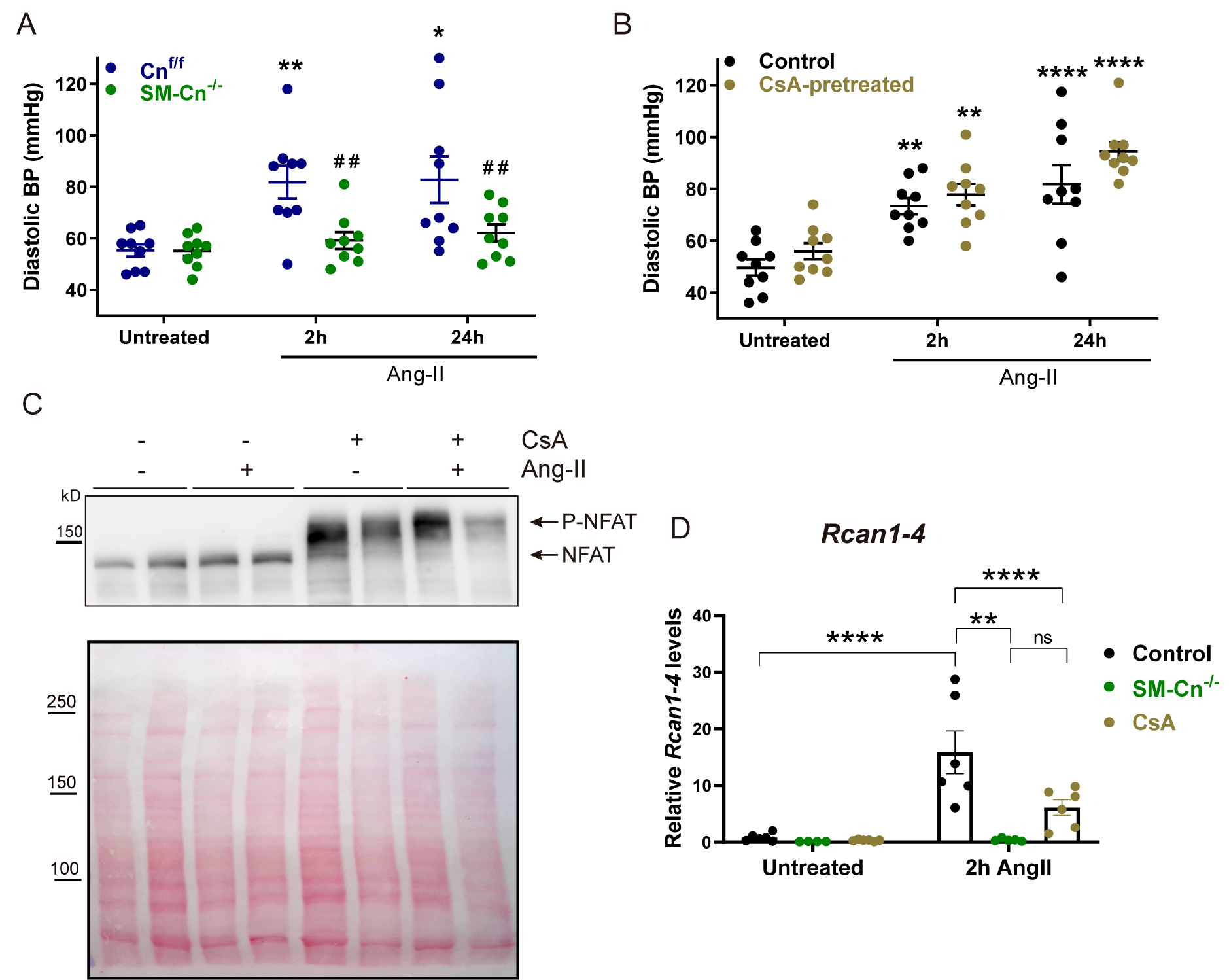

Supplement: S7 Fig — (A, B) Diastolic BP was measured at the indicated time points in the same Cn-Ctl, SM-Cn−/−, and non-pretreated and CsA-pretreated WT mice as in Fig 6B (A) and Fig 6D (B). Each data point denotes the BP value in an individual mouse, and the horizontal bars denote the mean (long bar) and the s.e.m. (n = 9 mice per group and per time point). ****p < 0.0001, **p < 0.01, *p < 0.05 vs. baseline; RM two-way ANOVA with Tukey’s post hoc test. ##p < 0.01 vs. 2 or 24 h Ang-II Cn-Ctl; RM two-way ANOVA with Šídák’s post hoc test. (C) Representative NFATc3 immunoblot of thymus extracts from mice treated as indicated. Hyper-phosphorylated NFATc3 (P-NFAT) and de-phosphorylated NFATc3 (NFAT) are indicated. Ponceau staining of the membrane is shown below. (D) Quantification of mRNA expression, as assessed by RT-qPCR, in extracts from the aorta of untreated control (3 Cn-Ctl plus 3 WT), SM-Cn−/− (n = 5), and CsA-pretreated (n = 6) mice and Ang-II-treated control (3 Cn-Ctl plus 3 WT), SM-Cn−/− (n = 5), and CsA-pretreated (n = 6) mice. Each data point denotes an individual mouse, and data in histograms are presented as mean ± s.e.m. **p < 0.01, ****p < 0.0001; two-way ANOVA with Šídák’s post hoc test. Underlying data can be found in S1 Data. (TIF) [file pbio.3003163.s007.tif]

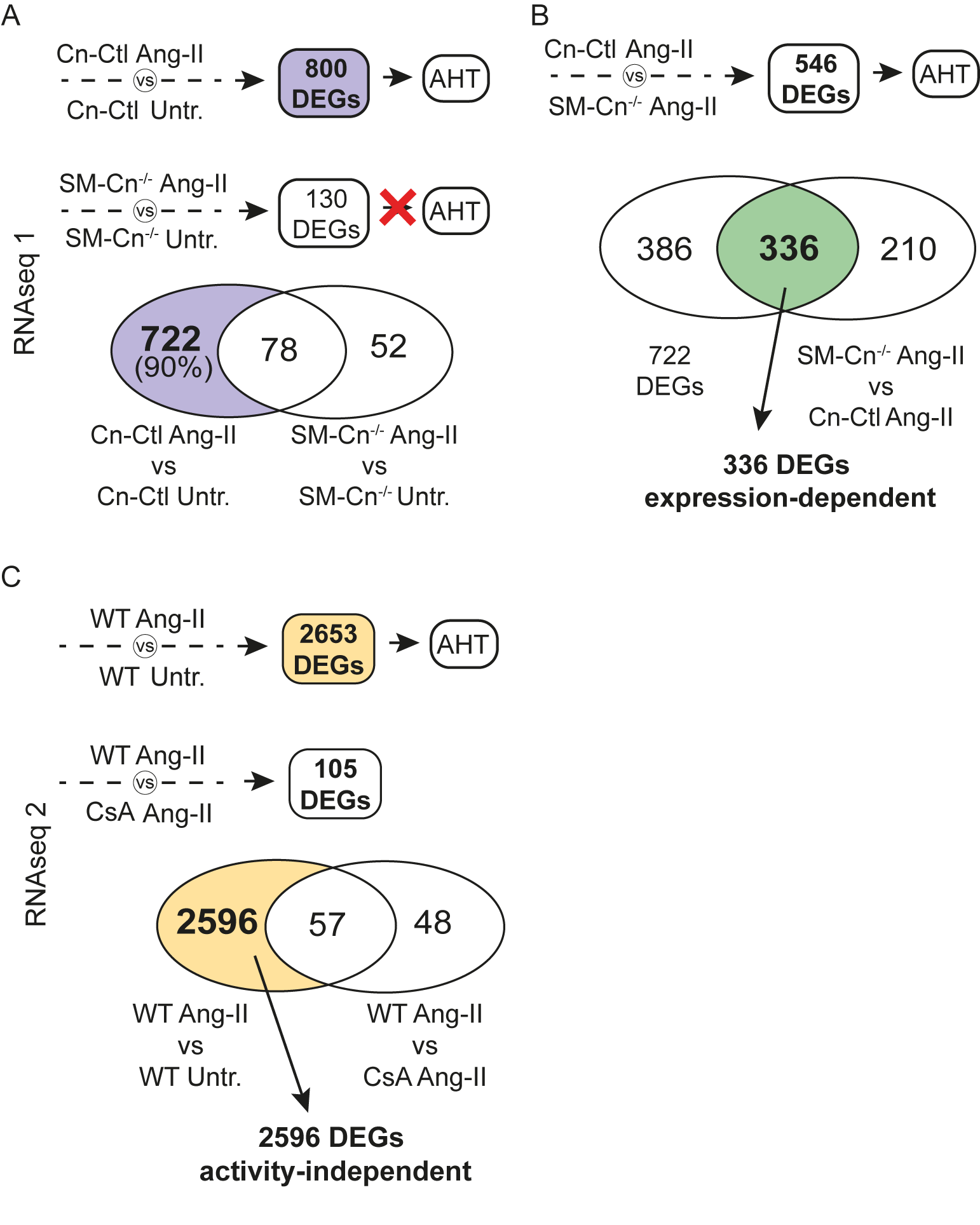

Supplement: S8 Fig — (A) 800 DEGs in Cn-Ctl mice treated with Ang-II for 2 h were selected as potential mediators of AHT, and 130 DEGs regulated in SM-Cn−/− mice treated with Ang-II for 2 h were excluded, leaving 722 potentially AHT-related genes, as indicated in the Venn diagram (below). (B) Of these genes, only those whose expression differed between the hypertensive condition (Cn-Ctl Ang-II 2 h) and the non-hypertensive condition (SM-Cn−/− Ang-II 2 h) were selected using a Venn diagram (below). The figure indicates the resulting 336 DEGs potentially involved in AHT and whose regulation is Cn-expression dependent. (C) 2,653 DEGs regulated in WT mice by treatment with Ang-II for 2 h were selected as potential mediators of AHT. Only those whose expression was similar in both hypertensive conditions (not showing differential expression between WT Ang-II 2 h and CsA Ang-II 2 h) and CsA-independent were selected using a Venn diagram (below). The figure indicates the resulting 2,596 DEGs potentially involved in AHT and whose regulation is independent of Cn phosphatase activity. (TIF) [file pbio.3003163.s008.tif]

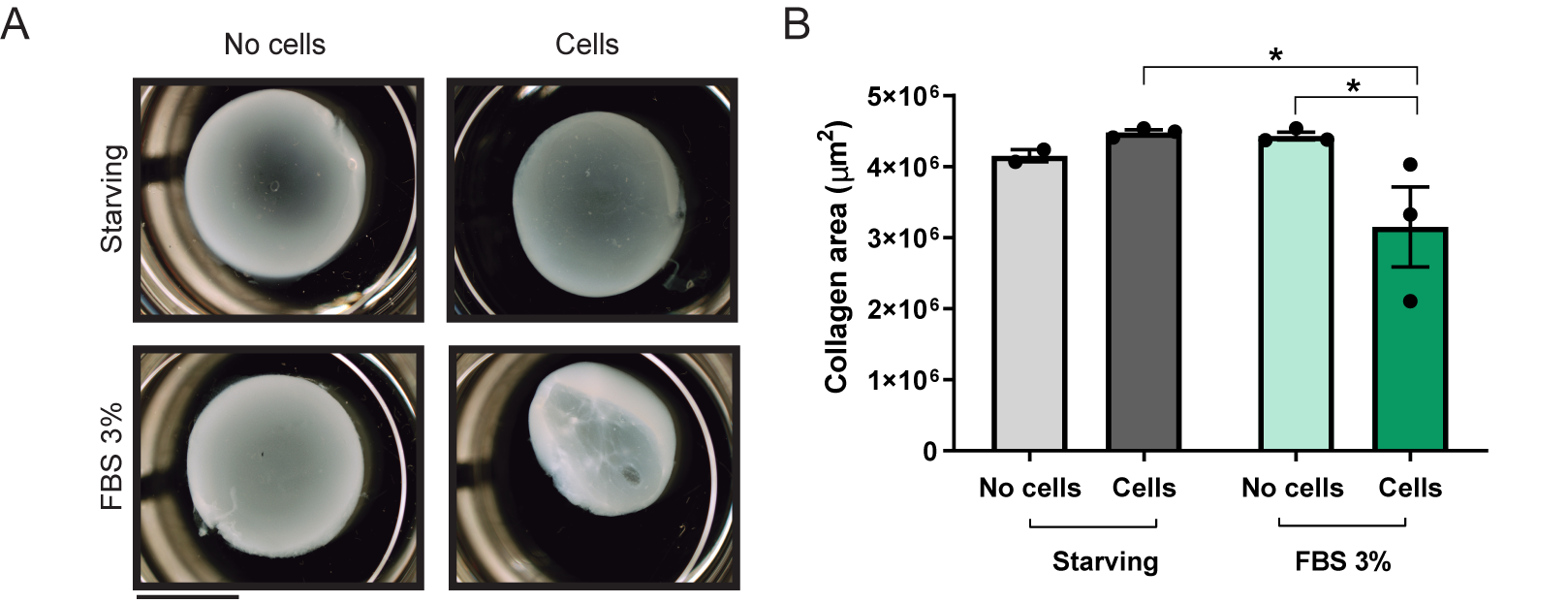

Supplement: S9 Fig — (A) Representative images from three independent experiments and (B) quantification of the surface area of fixed collagen gels in the absence or presence of cells stimulated as indicated. Each data point denotes the mean of each experiment, and data in histograms are presented as mean ± s.e.m. *p < 0.05 by two-way ANOVA with Šídák’s post hoc test. No cells without serum (n = 2), starved cells (n = 3), No cells with 3% FBS (n = 3), Cells with 3% FBS (n = 3). Underlying data can be found in S1 Data. (TIF) [file pbio.3003163.s009.tif]

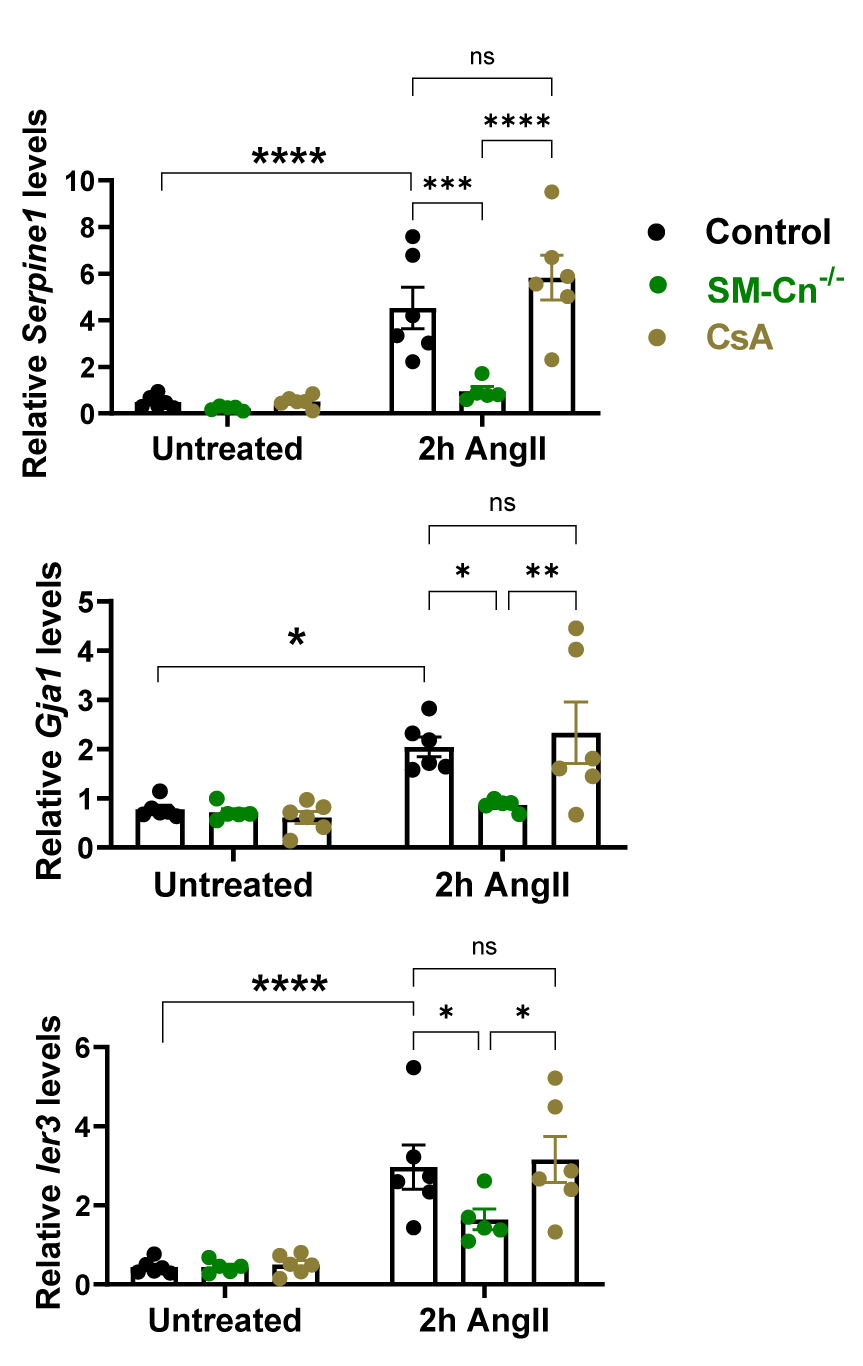

Supplement: S10 Fig — RT-qPCR analysis of mRNA expression in aortic extracts from untreated control (3 Cn-Ctl plus 3 WT), SM-Cn−/− (n = 5), and CsA-pretreated (n = 6) mice and from Ang-II-treated control (3 Cn-Ctl plus 3 WT), SM-Cn−/− (n = 5), and CsA-pretreated (n = 6) mice. Two-way ANOVA with Šídák post hoc test; ****p < 0.0001, ***p < 0.001, **p < 0.01, *p < 0.05. Underlying data can be found in S1 Data. (TIF) [file pbio.3003163.s010.tif]

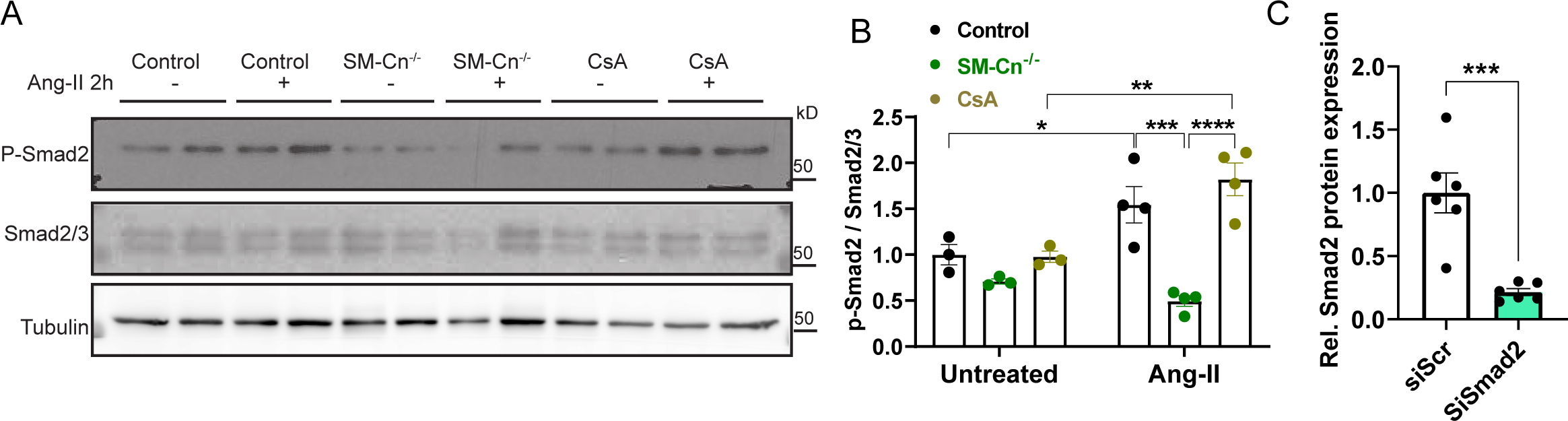

Supplement: S11 Fig — (A) Representative immunoblot analysis of phospho-Smad2, Smad2/3 and tubulin (loading control) and (B) quantification of their relative expression in protein extracts from untreated control (n = 3) and SM-Cn−/− (n = 3) mice; CsA-pretreated (n = 3) mice; and Ang-II-treated control (n = 4), SM-Cn−/− (n = 4), and CsA-pretreated (n = 4) mice. Molecular weights (kDa) are indicated. Each data point denotes an individual mouse, and data in histograms are presented as mean ± s.e.m. *p < 0.05, **p < 0.01, ***p < 0.001, ****p < 0.0001; two-way ANOVA with Šídák post hoc test. (C) Quantification of the relative expression in protein extracts from the representative immunoblot in Fig 8F (n = 3 independent experiments) with each data point representing an individual replicate. Data are presented as mean ± s.e.m. ***p < 0.001, unpaired Student t test. Underlying data can be found in S1 Data. (TIF) [file pbio.3003163.s011.tif]

A

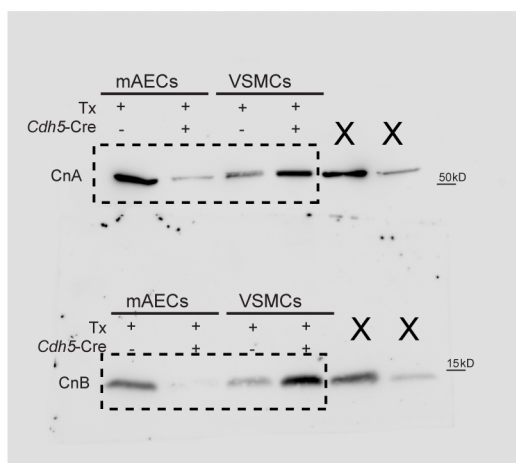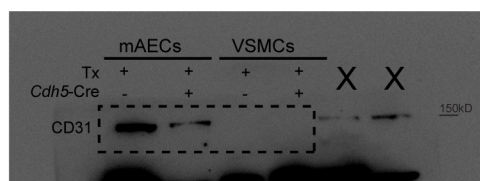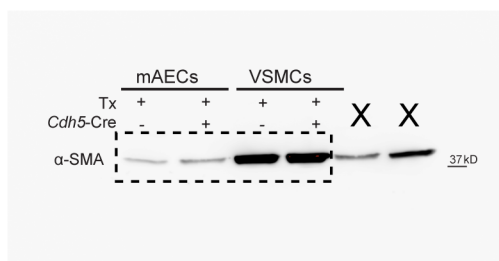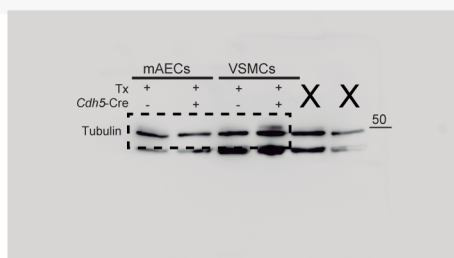

C

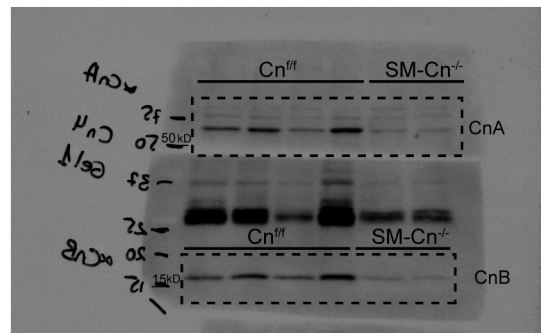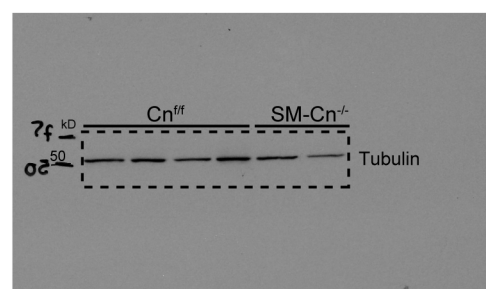

Figure 1

E

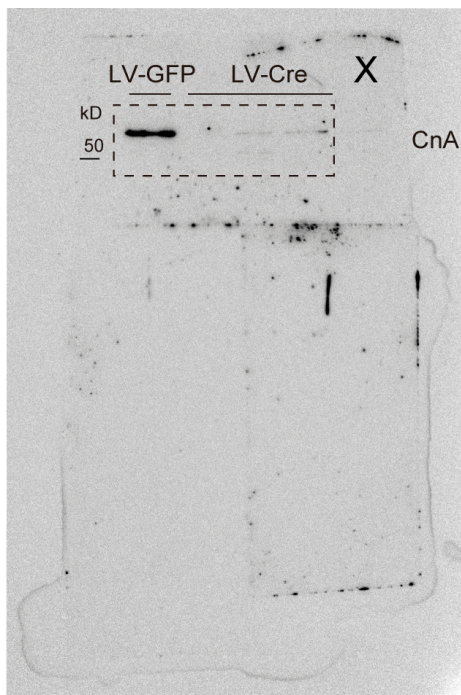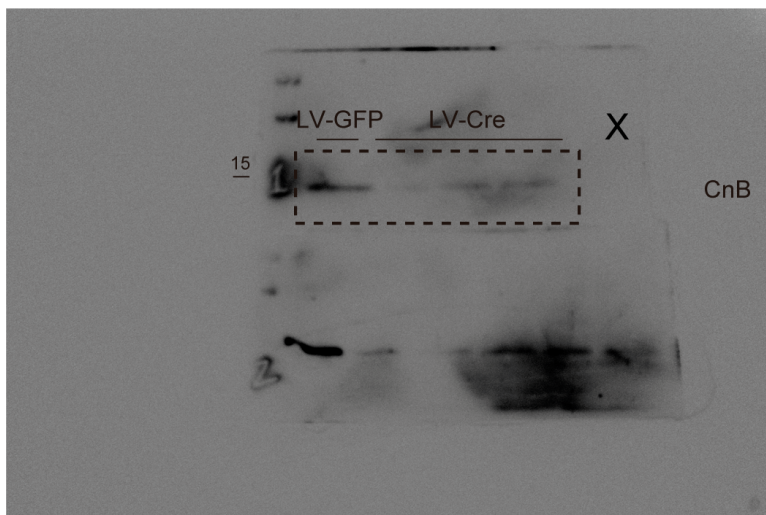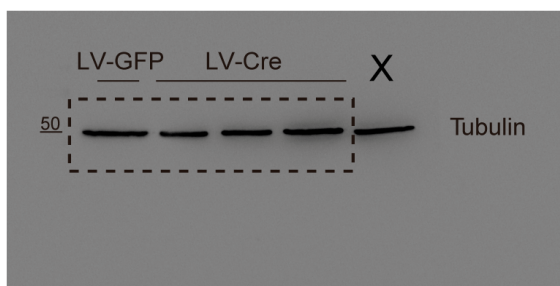

Figure 7

B

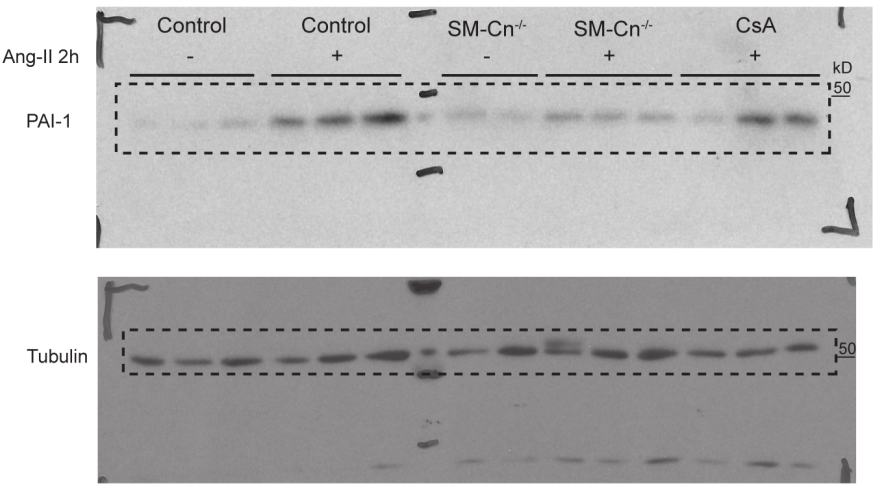

F

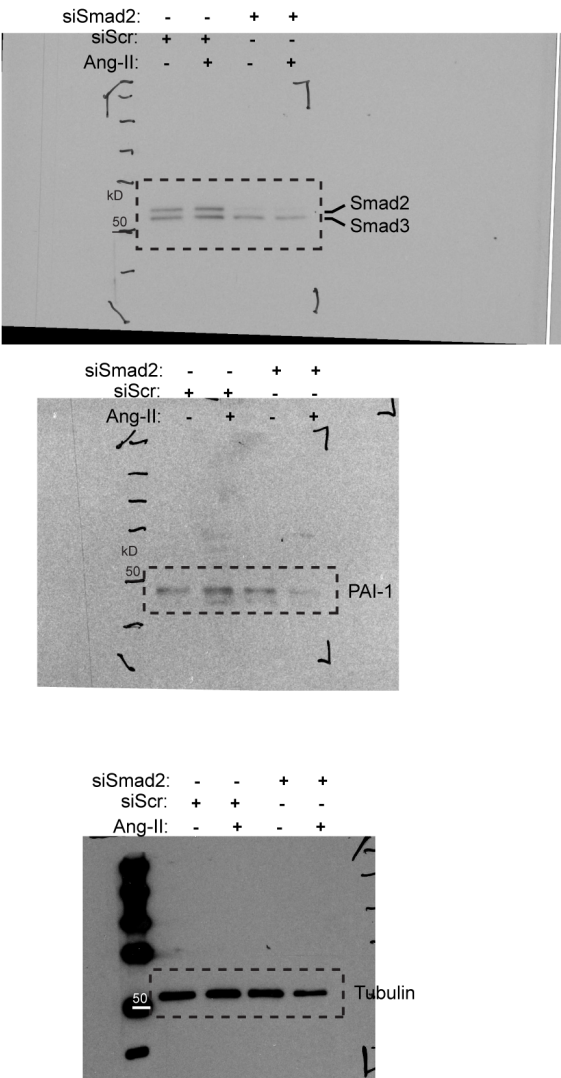

Figure 8

A

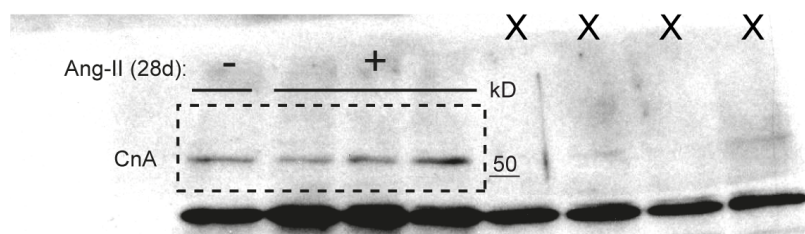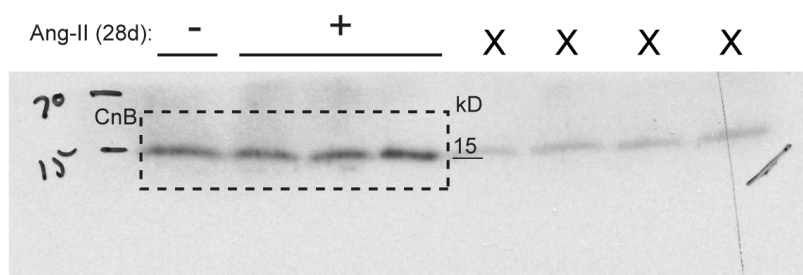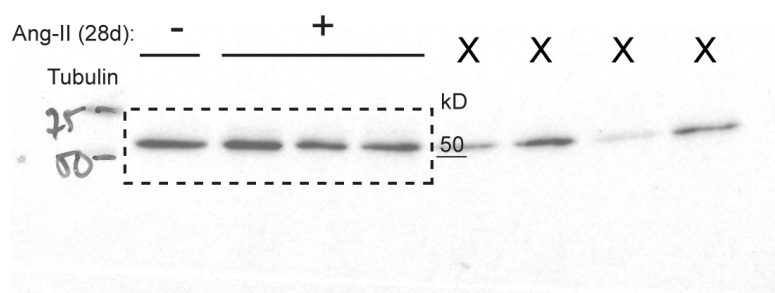

Supp. Figure 1

A

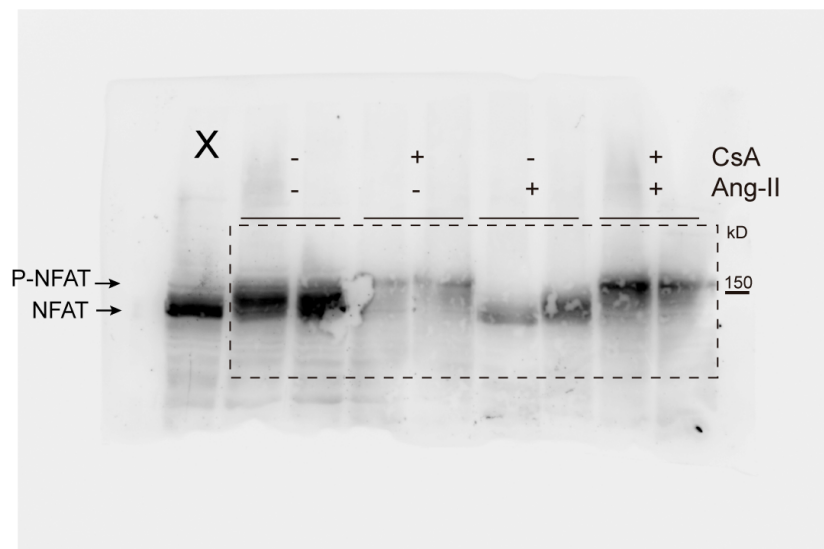

Supp. Figure 6

C

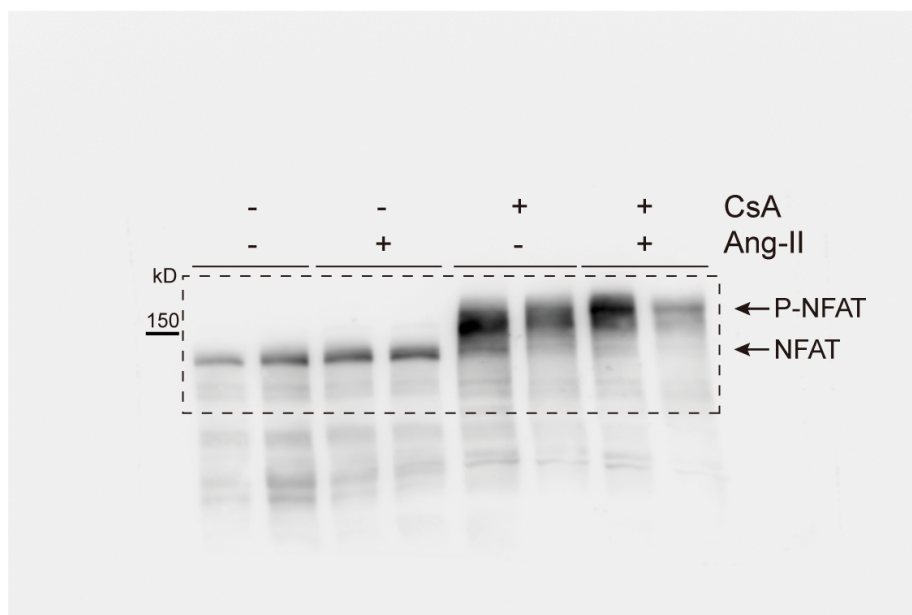

Supp. Figure 7

A

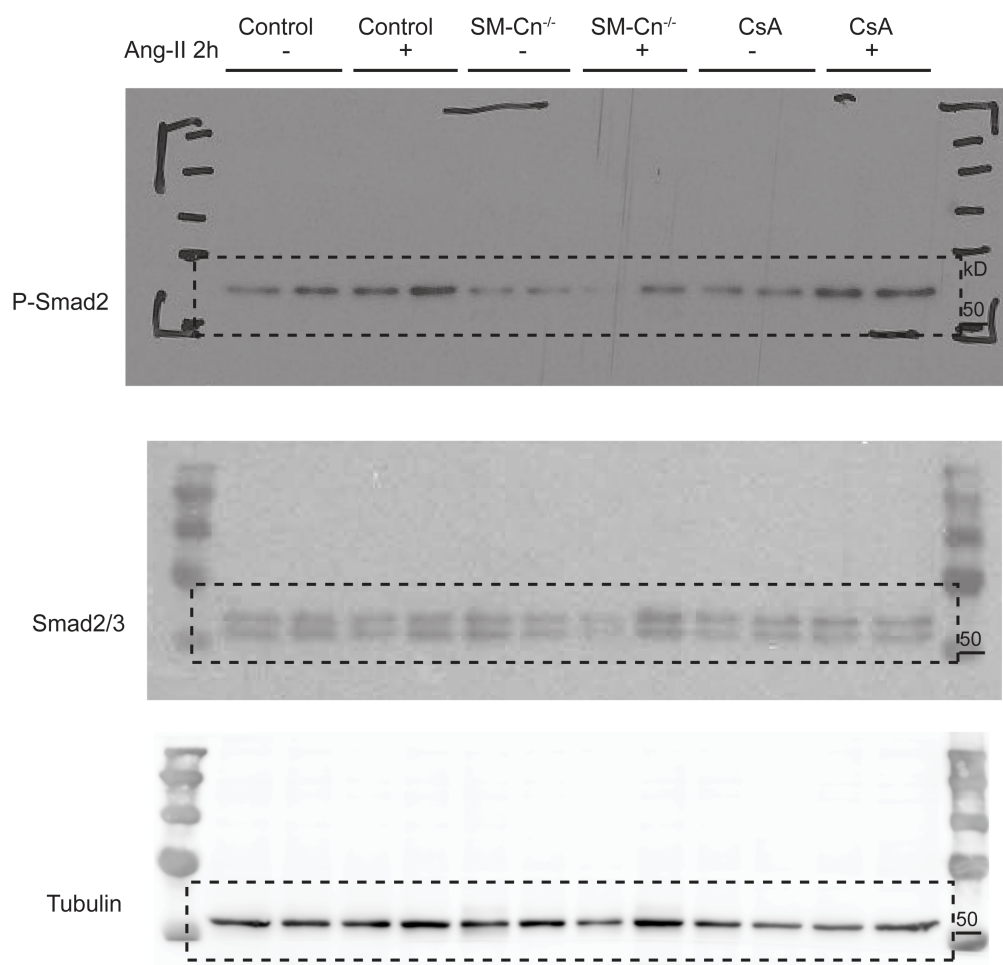

Supp. Figure 11

Supplement: S1 Raw Images — (PDF) [file pbio.3003163.s016.pdf]
